# Laser ablation microscopy reveals apical notch, apical dominance, and meristem regeneration dynamics in *Marchantia polymorpha*

**DOI:** 10.1073/pnas.2600460123

**Published:** 2026-06-22

**Authors:** Alan O. Marron

**Affiliations:** ^a^ Department of Plant Sciences, University of Cambridge, Cambridge CB2 3EA, United Kingdom

**Keywords:** Marchantia, meristem, apical dominance, regeneration, laser ablation microscopy

## Abstract

Meristems are the growth centers of plants and fundamental in understanding plant development, morphogenesis, and vegetative propagation. Across all plant groups, the phytohormone auxin controls meristem maintenance, represses the emergence of new meristems (apical dominance), and mediates cellular reprogramming when new meristems regenerate following removal of existing meristems. The liverwort Marchantia produces clonal propagules (gemmae) featuring two apical notches that develop into functional meristems. This presents a tractable experimental system to study meristem developmental biology. I used laser ablation microscopy to precisely disrupt cells in and around the developing premeristem in the apical notches of germinating gemma, finding that the first cell row is indispensable. Within this layer, a contiguous quorum of stem cells is required for activity. Apical notches reorientate in response to damage, demonstrating that the apical notch stem cells act as a communicating population. Feedback from the stem cell population is necessary to maintain notch activity and generate the notch apex. These experiments show communication between notches and regenerating meristems. The apical dominance signal represses cell division and requires both sources and sinks, features of auxin-mediated communication. Central regions of the gemma could transmit these apical dominance signals, but the tissues of the gemma periphery could not. I present a model of Marchantia gemma and apical notch organization, involving intra-, inter-, and extranotch communication. This provides a framework for further study of meristem formation, communication, and maintenance in Marchantia and improving knowledge of plant meristems more generally.

## Significance

Meristems are the growth centers of plants. Understanding how meristems function, how existing meristems stop new meristems emerging (apical dominance), and how cellular reprogramming regenerates meristems is central to understanding plant development. The model plant Marchantia offers a streamlined system whose evolutionary position makes its biology relevant to all plants. Marchantia apical notches display proliferative, regenerative, and dominance behavior typical of meristems. I used laser ablation to precisely disrupt cells in and around the developing premeristematic apical notch of the germinating Marchantia gemma, producing an improved model of Marchantia apical notch organization involving intra- and internotch communication characteristic of the phytohormone auxin. This will inform future Marchantia research and provides a simplified experimental framework to study more complex meristematic processes.

Cell division in plants occurs in meristematic regions. Meristems contain pluripotent stem cells which continually divide asymmetrically to produce a daughter and a meristematic cell. The cells at the center of the meristem divide slowly, while those at the periphery divide faster. This moves daughter cells outward as they mature and differentiate, while meristematic cells remain in the center, replenishing the stem cell pool. This spatial distinction between division and differentiation allows plant meristems to sustain growth and new organ formation simultaneously (1). It means that meristem size and spacing must be controlled for normal morphogenesis. All plant groups do this by means of positive and negative feedback loops involving signaling molecules such as CLE peptides and phytohormones. Disrupting the biosynthesis or transport of these signaling molecules leads to oversized meristems, incorrectly spaced meristems, or loss of meristem function (2, 3).

Perhaps the most important of these signaling molecules is the phytohormone auxin. In vascular plants, auxin is produced in the shoot apical meristem (SAM) and flows outward by polar transport, where it is involved in controlling cell expansion and differentiation (4, 5). Secondary meristem formation is induced by the formation of local auxin maxima and minima arising out of auxin gradients extending from the apical meristems (5). Apically derived auxin inhibits growth of secondary meristems in the buds: this is apical dominance (3). If the SAM is damaged or destroyed, the flow of auxin is disrupted, apical dominance is lifted and these meristems are released from dormancy and begin growing (6). Plants have the capacity to regenerate meristems in response to localized wounding, which changes the auxin:cytokinin ratio around the damage site. In vascular plants, this causes cells from certain tissue types (e.g., embryonic tissue, pericycle) to lose their previous identity, respecify their cell fate and become meristematic tissue (5, 7). This cellular reprogramming to acquire meristem identity involves changes in gene expression, e.g., activation of WIND, WUS, WOX, and PLT transcription factors (7). Bryophytes, such as the liverwort *Marchantia polymorpha*, have massive capacity for regeneration: All parts of the plant, and even single cells, can regenerate meristems de novo that eventually produce full plants (8–13).

**Fig. 1.**
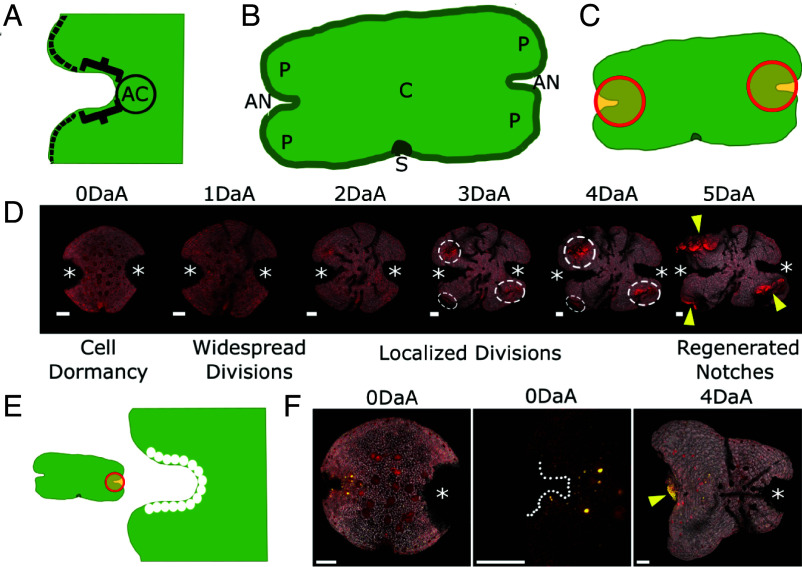
Laser ablation shows that the first row of cells in the apical notch are indispensable for meristematic activity. (*A*) Apical notch schematic. AC marks the apical cell at the notch apex or center, flanked by subapical cells (brackets). These cells comprise the first row of cells. Further outward the first row of cells is margin tissue (dashed line) (9). (*B*) Gemma schematic showing two apical notches (AN) at either end, the peripheral regions (P) to the sides and the central region (C) behind the notches. The stalk scar (S) is where the gemma was attached to the parent thallus within the gemma cup. (*C*) Schematic of the ablation pattern (orange bounded by red) for complete excision of all notches. (*D*) Time course of a gemma fragment expressing an mScarlet cell membrane marker (p5-UcE2:mSc-Lti6b) following complete notch excision. The four main phases of meristem regeneration are described. Excised notches are marked by asterisks. Cell division can be seen by increased density of red signal, localized patches of cell division are marked by dashed circles and regenerated notches marked with yellow arrows. Images were acquired at the indicated time-points after apical notch removal (days after ablation, DaA). (*E*) Schematic of the ablation patterns used in the fine scale gemma ablation experiment. Orange bounded by red denotes excised notch, white circles indicate ablated cells. (*F*) shows, from *Left* to *Right*, the 0DaA gemma (asterisk marking excised notch), 0DaA notch close up (ablated cells marked by white circles) and the 4DaA gemma (the yellow arrow marks regenerated notch). Ablating the first row of cells in the apical notch is sufficient to induce meristem regeneration, which occurs just behind the ablated notch. Gemma shown is from the enhancer trap apical notch/meristem marker line ET239-P153. See *SI Appendix,* Fig. S2*B* for the full time series. Confocal images show membrane marker in red, nuclear mVenus enhancer trap signal in yellow and chlorophyll autofluorescence in gray. (Scale bars, 100 μm.)

Marchantia grows as a dominant haploid gametophyte thallus, from which develops a diploid sporophyte. Meristems are present in both generations, with some genetic pathways common to both, but other pathways specific to one generation or the other (14–16). The sporophyte lacks an apical meristem, instead growing by cell division along its length (17). The gametophyte thallus has apical meristems located in concave apical notches (Fig. 1*A*), which are the loci of cell proliferation. The apical notch consists of a central apical cell with four cutting faces, surrounded by dorsal and ventral merophytes, plus lateral subapical cells (18, 19). Around each apical notch is a region of cell differentiation. As cells move outward away from the meristem, division rates reduce, and cell expansion increases. The apical meristems appear early in sporeling development, and their emergence marks the transition from an undifferentiated callus-like protonema into a prothallus, which goes on to form the thallus (9, 20). As the thallus grows the apical notch meristems enlarge and bifurcate, thus producing new thallus branches by dichotomous branching (21). The thallus also forms dorsal outgrowths called gemma cups, within which grow vegetative propagules called gemmae (22). Gemmae have two apical notches located at either end (Fig. 1*B*). Gemmae remain dormant until removal from the gemma cup, at which point they germinate. Immediately after germination, the apical notches are in effect premeristems, with dorso-ventral and left–right symmetry. This could be considered equivalent to the developing SAM of the late torpedo stage angiosperm embryo, where a stem-cell containing notch, but not yet a morphologically mature meristem, has formed between two cotyledons (23). Over the course of the next 5 d meristem development continues in the notch, producing a complex, mature meristem with only left–right symmetry. This can be seen from changes in expression of genes like Mp*YUCCA2*, Mp*C3HDZ,* and Mp*C4HDZ* within the apical notch and across the growing gemma (9, 24).

The gametophyte apical notch meristem demonstrates apical dominance dynamics and lateral inhibition (25). Apical notches prevent the emergence of new active meristems (outside of dichotomous branching) and repress dormant meristems, such as gemmae within the gemma cup (26) or shaded apical notches (21). If active apical notches are removed, then meristems kept dormant by lateral inhibition start growing (27). If all apical notches are removed then apical dominance is lifted and meristems regenerate de novo (8, 11). Auxin has long been known to be involved in meristem regeneration in Marchantia (25, 27–30). Auxin is produced in the apical notch and is exported to the rest of the thallus, where it inhibits formation of new meristematic regions (i.e., apical dominance). Removing apical notches removes these auxin sources. Initially auxin levels drop in the remaining plant, and then rise as new meristems emerge (8).

Developing better insights into how meristems function is key to understanding plant development and producing beneficial new morphologies (2, 3). Understanding the molecular mechanisms of cellular reprogramming to convert different cell types into premeristematic and meristematic tissue is important in vegetative propagation of agricultural crops and offers an avenue to develop new transgenic methods (7, 31). Considerable progress on understanding apical meristems has been made from study of angiosperms, particularly *Arabidopsis* (32), however these systems have limitations, such as difficult access for imaging (particularly live imaging) (33) and the complexity of the gene networks involved (2, 4).

Although the premeristem and meristem of the Marchantia apical notch has received relatively little attention compared to angiosperm meristems (16, 34) it offers a promising opportunity to gain insights into meristematic tissues generally. The key advantage of Marchantia as an experimental system for plant biology is its streamlined nature. For many gene families the Marchantia genome has fewer copies compared to other model plants like *Arabidopsis* or *Physcomitrium* (16, 35), notably the minimal set of components related to auxin biosynthesis, transport, and signaling (35–40). It is highly experimentally tractable, with fast growth rates and well-established transgenic protocols (41, 42). The gemma is well suited for developmental experiments, since it grows in a stereotypical pattern. Crucially, gemmae have open development where almost all of the cells and tissues are easily accessible for microscopy without the need for dissection, sectioning, or clearing (41).

Marchantia, as a bryophyte, occupies an important position in the land plant phylogeny (15, 43, 44). Although the thalloid gametophyte is not homologous to the dominant diploid sporophyte of vascular plants, phylogenetic analyses and molecular research finds that the apical notch (pre)meristem shares many regulatory modules in common with angiosperm apical meristems (14, 45–50). The SAM model is thought to be how meristems operated in the first land plants (51), making findings from Marchantia applicable to all plant meristems (52). Inter- and intra-apical notch signaling in the Marchantia gemma allows study of premeristem and meristem communication, and of the gene networks regulating meristem morphology (8, 45, 46, 53, 54). Meristem regeneration in gemmae provides an opportunity to investigate cellular reprogramming and how emerging premeristematic regions interact. This can provide insights into how Marchantia specifies new meristems during spore germination (9, 20, 55), gemma cup development (22), and apical notch bifurcation (21, 45), processes relevant to vegetative reproduction and lateral branching in crop species. In addition to its importance in plant evo-devo studies, Marchantia holds great promise as a chassis for synthetic biology (41, 56), and greater understanding of Marchantia meristems will further this research.

I used laser ablation and transgenic marker lines to make precise disruptions in the apical notches of gemmae and observed how this altered cell proliferation and apical dominance (55, 57, 58). My results point toward a model with a dynamic population of stem cells communicating within the apical notch and with the rest of the gemma. Only certain regions of the gemma can conduct these communication signals, and a balance of auxin sources and sinks is required to sustain meristematic activity. Disruption of this communication and balance induces new meristems to emerge, or causes existing apical notches to cease cell division.

## Results

### Laser Ablation of the Apical Notch Induces Meristem Regeneration.

Marchantia plants expressing a plasma membrane marker fused to mScarlet driven by a ubiquitous promoter (p5-UcE2:mSc-Lti6b) allowed the visualization of new cell membranes and cell division. Laser ablation microscopy was used to remove both notches of a 0 d post germination (dpg) gemma (Fig. 1*C*) and the membrane marker line used to track meristem regeneration (Fig. 1*D*). This background experiment confirmed previous findings (59), that there are four distinct phases of regeneration in Marchantia. In the first ~24 h little or no cell division occurs. Between 24 to 48 h after ablation, there is rapid, widespread cell division across the gemma. After ~48 h, cell division becomes restricted to localized patches of cell division (dashed circles). Approximately 5 d after ablation (DaA) meristem regeneration has progressed to formation of apical notches (yellow arrows).

A collection of enhancer trap lines were used to monitor apical notch activity and regeneration. The genes marked are not yet identified but these lines were all previously established to mark the apical notch/meristem region via nuclear mVenus signal (9) Control ablation experiments demonstrated that the location and progress of meristem regeneration could be monitored using the enhancer trap signal (*SI Appendix,* Fig. S1). Nuclear mVenus marks patches of localized cell division, only persists in actively dividing regions and is maintained in active apical notches. Loss of marker signal indicates loss of apical notch identity and cell proliferation activity.

Examination of multiple gemmae with both notches completely removed (ablation pattern in Fig. 1*C*) did not find any clear predictability as to which regions would go on to form new apical notches, nor how many patches of localized cell division or regenerated notches there would be. The exception to this is the stalk scar region, which was never observed to initiate cell divisions.

Laser ablation microscopy can make fine scale perturbations in the apical notch to see how much tissue must be removed to induce meristem regeneration. One apical notch was entirely excised and in the other notch a number of rows of cells were ablated. Ablating one cell row deep into the apical notch was sufficient to induce meristem regeneration (Fig. 1 *E* and *F* and *SI Appendix,* Fig. S2 *A* and *B*). Notch organization was disrupted following ablation, with the area expanding outward and meristem regeneration always occurring in the region behind the ablated row of cells (yellow arrow, Fig. 1*F* and *SI Appendix,* Fig. S2*B*). Ablating the first three rows of cells was required for meristem regeneration to be induced elsewhere in the gemma, away from the notch (*SI Appendix,* Fig. S2 *C* and *D*). These background investigations established the first row of apical notch cells as the focus for subsequent laser ablation experiments.

### Laser Ablation Demonstrates Interactions between Apical Notches.

The current Marchantia literature centers around a model where, following germination, each apical notch contains a single apical cell. This divides, giving rise to lateral subapical cells, which comprise the rest of the first cell row in the apical notch. However until recently there has been little experimental detail on how these cell types are specified, how they interact with each other or with other apical notches (54, 55). I interrogated this in more detail by ablating specific parts of the crucial first row of apical notch cells in the developing premeristem. A flow chart of the outcomes of these experiments is provided in *SI Appendix,* Fig. S3.

Ablating one side of the apical notch did not disrupt meristematic activity (i.e., cell proliferation and apical dominance), irrespective of whether or not there was another intact apical notch present in that gemma (*SI Appendix,* Fig. S4 *A* and *B*). However, leaving only a few apical notch cells intact on one side disrupted meristematic activity. If the other apical notch in the gemma was intact (*SI Appendix,* Fig. S4*C*) then the partially ablated notch became inactive due to apical dominance from the intact notch. If the other notch was excised (*SI Appendix,* Fig. S4*D*), the remaining cells in the partially ablated notch were insufficient to sustain apical dominance, and meristem regeneration occurred elsewhere in the gemma fragment. When a few apical notch cells were left intact on both sides of the notch then this partially ablated notch always lost meristematic activity (*SI Appendix,* Fig. S4 *E* and *F*). Notches with multiple small subpopulations of intact cells lost meristematic activity, even if there were more intact cells in total compared to functional notches where the intact cells comprised a contiguous row.

**Fig. 2.**
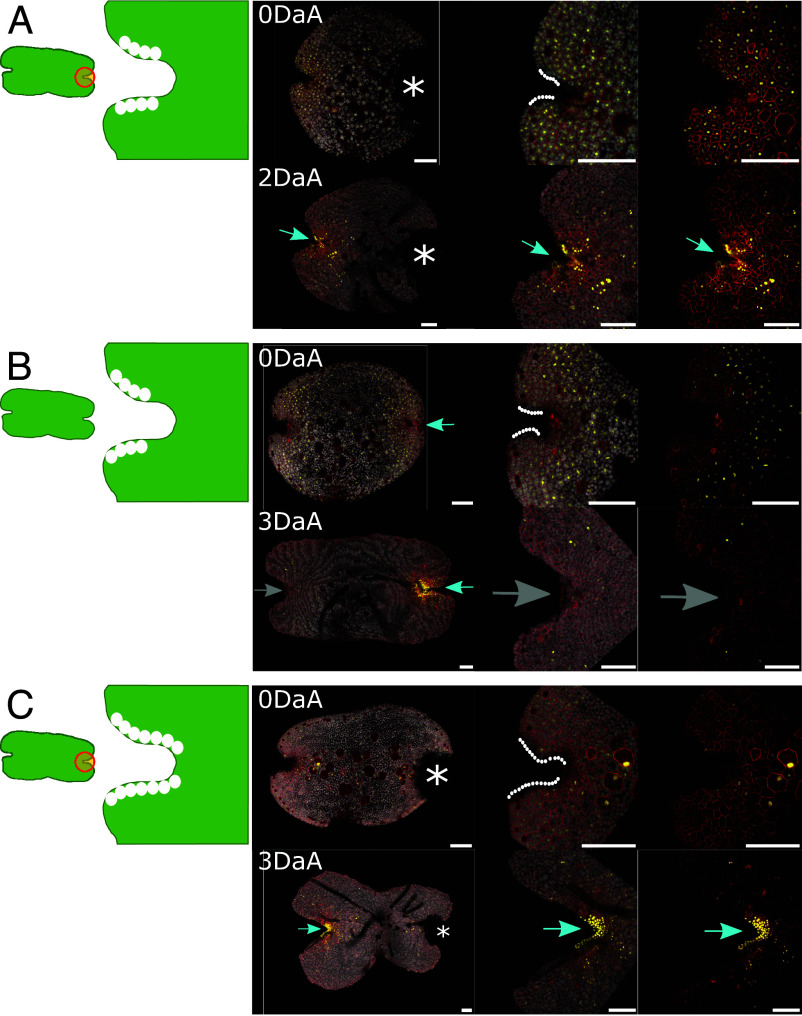
The notch apex, containing the apical cell, can sustain meristematic activity only if the other apical notch is excised. Ablation of the notch sides, leaving the notch apex (including the apical cell) intact, did not disrupt meristematic activity if the other apical notch in the gemma was excised (*A*), but when the other apical notch was intact then meristematic activity ceased (*B*). (*C*) Meristematic activity continued even if few cells were left intact at the apex if the other notch was excised. In each subfigure, a schematic of the ablation pattern is shown with white circles marking the location of ablated cells and orange bounded by red denoting excised tissue. Time courses are presented in the format whole gemma, notch close up, notch close-up without chlorophyll autofluorescence channel. Asterisks mark excised notches, blue arrows denote active apical notches, gray arrows mark apical notches that have lost meristematic activity. In the 0DaA images, white circles mark ablated cells. The enhancer trap apical notch/meristem marker lines used were ET239-P21 (*A* and *B*) and ET239-P153 (*C*). (Scale bars, 100 µm.)

When both sides of the apical notch were ablated, but a few cells at the apex (including the presumed apical cell) were left intact, then the outcome depended on the status of the other notch in the gemma. If the other notch was excised (Fig. 2*A*), then the intact apex continued to function as an apical notch (blue arrow) and retained apical dominance as no new patches of cell division emerged elsewhere. When the other notch in the gemma was intact (Fig. 2*B*), then the intact apex alone could not sustain meristematic activity (gray arrow), even if more cells remained compared to cases where meristematic activity continued with the other notch excised (e.g., Fig. 2*C*).

**Fig. 3.**
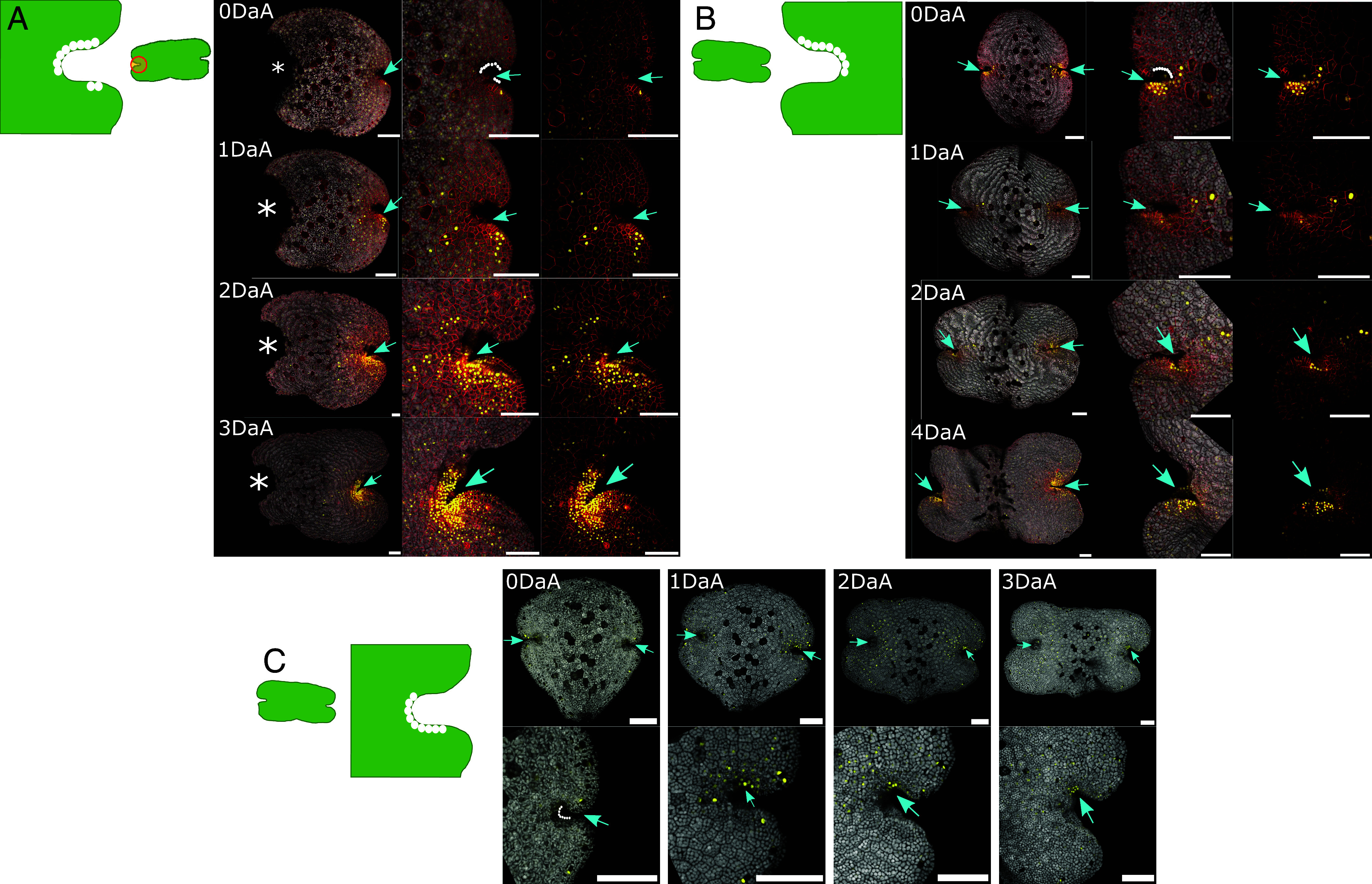
Notch reorientation can occur after partial ablation of the notch apex, independent of the status of the other apical notch in the gemma. Partial notch ablation, such that the intact stem cell subpopulation spans the apex and one side of the notch, results in reorientation of the apical notch. The notch changes shape, forming a new apex whose location corresponds to where the center of the subpopulation of intact cells was originally. Reorientation occurs whether the other notch in the gemma was excised (*A*) or intact (*B* and *C*). Meristematic activity continued during reorientation, as indicated by mVenus expression in enhancer trap lines (*A* and *B*). Mp*YUCCA2* promoter activity (*C*) shows that the new apex becomes the focus of auxin production in the reorientated notch. In each subfigure, a schematic of the ablation pattern is shown with white circles marking the location of ablated cells and orange bounded by red denoting excised tissue. Time courses in *A* and *B* are given in the format whole gemma, notch close up, notch close-up without chlorophyll autofluorescence channel. Asterisks mark excised notches; blue arrows denote active apical notches. In the 0DaA images, white circles mark ablated cells. *A* and *B* show the enhancer trap apical notch/meristem marker lines ET239-P21; C shows a Mp*YUC2* promoter marker line with mTurquoise signal in yellow. (Scale bars, 100 µm.)

Parts of the notch spanning the apex and sides were ablated, including where the apical cell is located (Fig. 3). When some of the apical notch cells were ablated, but a sufficient subpopulation remained as a contiguous row, then not only did the notch continue proliferative activity (blue arrows), but over the next 48 h its growth pattern changed. By 3DaA, what was previously the center of the intact subpopulation of apical notch cells has become the notch apex, i.e., the notch had reorientated. Ablation experiments on Mp*YUC2* promoter marker line gemmae (Fig. 3*C*) found that promoter activity continued during reorientation and that at 3DaA the reorientated apex was the focus of Mp*YUC2* expression, and therefore auxin biosynthesis. This shows that reorientation is not just superficial, but that the mechanistic activity of the apical notch also shifts position.

Reorientation occurred irrespective of where the apex of the original notch had been before ablation and did not appear to be influenced by whether the original center of the notch (i.e., the location of the apical cell) was ablated or not. During the first 48 h after ablation, no new regions of cell division appeared (if the other apical notch in the gemma had been removed), nor were reorientating notches subject to apical dominance-related inhibition (if the other notch in the gemma was intact). There were no changes in Mp*YUC2* promoter activity in the partially ablated notch or across the rest of the gemma that might indicate an interruption or major spatial reorganization of auxin biosynthesis. Notch reorientation occurred even when gemmae were grown on media containing 3 µM 1-naphthylacetic acid (1-NAA) (*SI Appendix,* Fig. S5), a pharmacological inhibitor of regeneration (8). This indicates that reorientating notches continued to be apically dominant and that the morphological changes involved in notch reorientation were not due to de novo meristem regeneration.

### Meristem Regeneration Involves Communication between Regions of Cell Division.

**Fig. 4.**
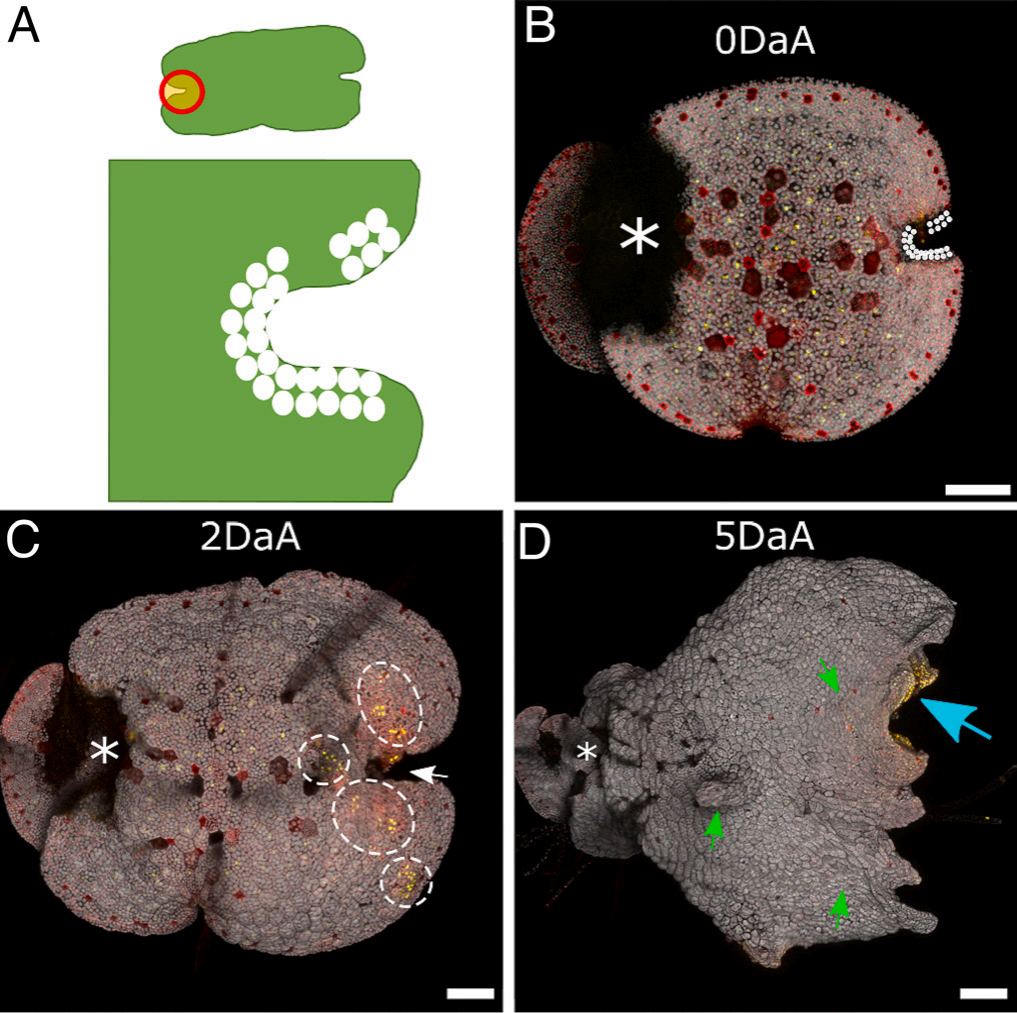
Time course demonstrating reestablishment of apical dominance by a partially ablated apical notch. (*A*) Schematic of the ablation pattern used. White circles mark ablated cells, orange bounded by red marks excised tissue. (*B*) shows the gemma immediately after laser ablation. White circles mark the position of ablated cells, the asterisk indicates the entirely excised apical notch. (*C*) At 2DaA patches of localized cell division and marker signal appeared (dashed circles), even though the original, partially ablated notch still displayed marker signal (white arrow). (*D*) By 5DaA, the original partially ablated notch had meristematic activity (blue arrow), as indicated by the presence of dense marker signal, and the apex of this notch had reorientated. The areas that were patches of cell division had no marker signal (i.e., no cell division activity) and remained as protrusions on the gemma surface (green arrows). The complete time course is given in *SI Appendix,* Fig. S6. The enhancer trap apical notch/meristem marker line used was ET239-P125. (Scale bars, 100 µm.)

Fig. 4 and *SI Appendix,* Fig. S6 show a gemma ablated so that one apical notch was entirely excised and in the other notch a short, contiguous subpopulation of cells was left intact. Initially, the rest of the gemma was released from apical dominance and proceeded to the early phases of regeneration. By 2DaA there were regenerating patches of localized cell division with dense enhancer trap marker signal (dashed circles, Fig. 4*C*). Simultaneously, the partially ablated apical notch also recommenced cell division (white arrow, Fig. 4*C*). By 3DaA this region was the most prominent area of marker signal, though other patches of signal were visible (*SI Appendix,* Fig. S6*D*). Five days after ablation (Fig. 4*D* and *SI Appendix,* Fig. S6*F*), the notch that was originally partially ablated had the characteristic apical notch morphology and was actively proliferating (blue arrow). It had reestablished apical dominance, and the other patches stopped division and meristem regeneration (indicated by loss of marker signal, green arrows). However, these areas progressed considerably through meristem regeneration, with sufficient cell divisions to produce protrusions on the thallus.

### Directional Flow of Signal Out from the Apical Notch Causes Apical Dominance.

The apical dominance signal produced at the apical notch must flow outward in order to influence the rest of the gemma. Tissue was ablated in different orientations to the apical notch to see if the resulting incisions could interrupt the flow of signal. Incisions made perpendicular to the notch had no obvious effect (*SI Appendix,* Fig. S7 *A*–*E*). Incisions made parallel to the notch (*SI Appendix,* Fig. S7 *F*–*O*) resulted in more cell division proximal to the incision and increased cell expansion distal to the incision. This effect was not seen when the portion of the apical notch immediately proximal to the parallel incision was ablated; instead cells on both sides of the incision ceased dividing and expanded.

**Fig. 5.**
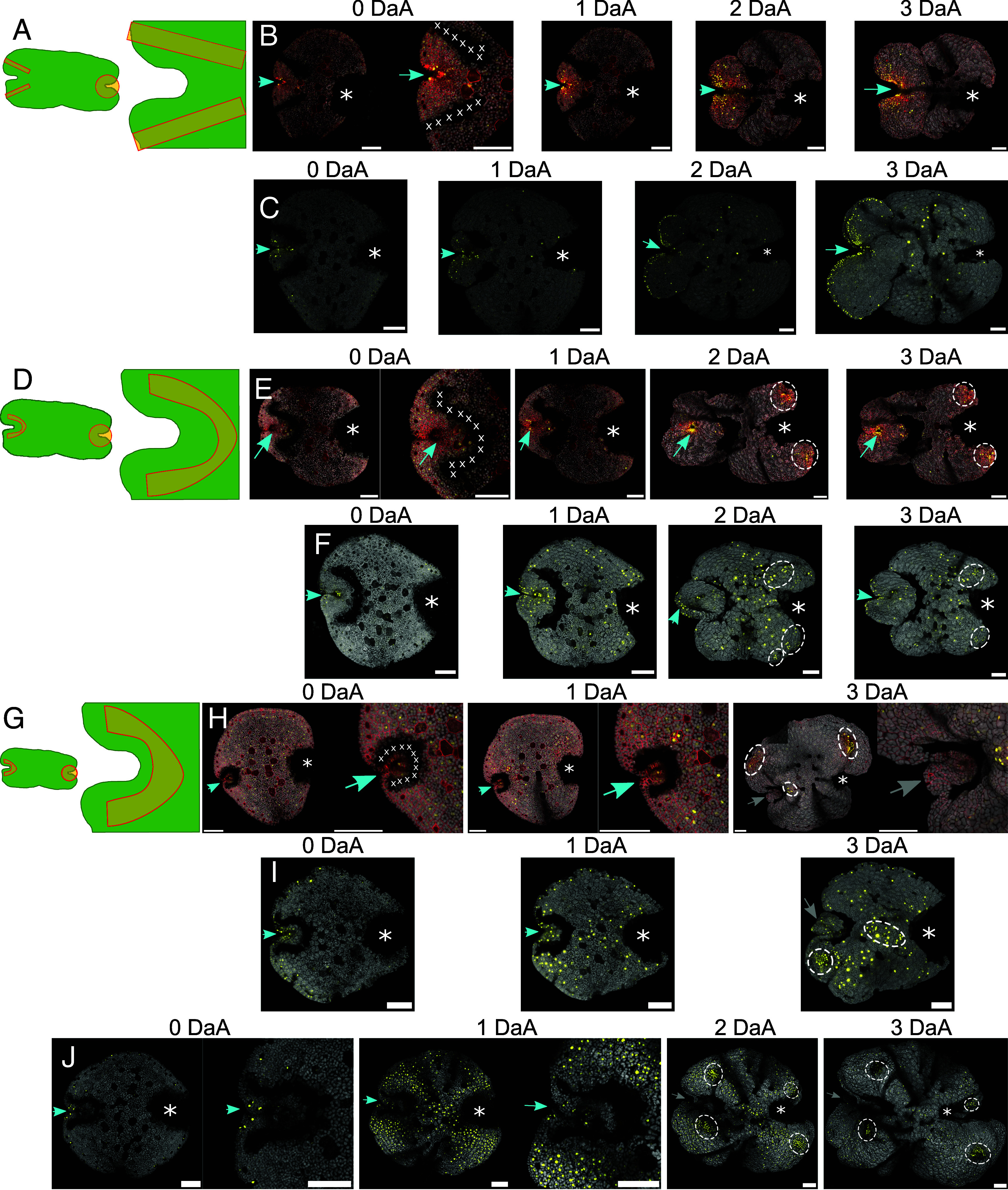
Ablating tissue around the apical notch demonstrates that central regions of the gemma can transport apical dominance signal, but peripheral regions cannot conduct apical dominance signal. (*A*) Schematic of the ablation pattern used in time courses (*B*) and (*C*). The intact apical notch maintained meristematic activity (blue arrows). No meristem regeneration occurred in the gemma fragment attached to this notch by a central connection. (*C*) shows a marker line for Mp*PIN1* promoter activity. There is signal at the notch and the gemma edge but little in the center of the gemma, and none in the central connection itself. (*D*) Schematic of the ablation pattern used in time courses (*E* and *F*). The apical notch remained active (blue arrows) however meristem regeneration (dashed circles) occurred in the gemma fragment attached to this notch by the peripheral connections. In (*E*) this was marked by the appearance of new enhancer trap mVenus marker signal, while in (*F*) this was indicated by proliferation of Mp*PIN1* promoter signal throughout the gemma, and later by clusters of Mp*PIN1* promoter signal. Though signal was visible in the peripheral connections early on in time course (*F*), this connection apparently did not conduct apical dominance signal from the intact notch. (*G*) Schematic of the ablation pattern used in time courses (*H*–*J*), where the gemma was ablated to leave only a small amount of tissue surrounding the intact apical notch. By 3DaA, regenerating meristems emerged (dashed circles). The intact apical notch was initially active, indicated by enhancer trap marker expression (blue arrows in *H*) but by 3DaA this notch was inactive (gray arrows), with the cells losing marker signal, ceasing division and expanding. In the Mp*PIN1* (*I*) and Mp*YUC2* (*J*) promoter marker line time courses, loss of signal was observed at the inactive notch (gray arrows). At 1DaA there is widespread Mp*YUC2* promoter activity across the gemma fragment, but this becomes restricted to progressively smaller regions as meristem regeneration proceeds (*J*). The asterisk marks entirely excised notch, blue arrows denote active apical notches, gray arrows denote inactive apical notches, dashed circles mark patches of localized cell division. In schematics (*A*, *D*, and *G*) X marks incision ablations, orange bounded in red denotes excised tissue. Gemmae shown are from the enhancer trap apical notch/meristem marker line ET239-P153 (*B* and *E*), ET-239-P125 (*H*), Mp*PIN1* promoter marker line with mVenus signal in yellow (*C*, *F*, and *I*) or Mp*YUC2* promoter marker line (*J*). (Scale bars, 100 µm.)

More extensive incisions found that the type of connection to the rest of the gemma was crucial for transport of the apical dominance signal (Fig. 5). Connections formed of tissue from the central part of the gemma (Fig. 5 *A*–*C*) communicated the repressive signal from the active apical notch (Fig. 5, blue arrows) and did not see meristem regeneration elsewhere. This was the case even when the connecting tissue was only a few cells wide.

In contrast, connections formed from tissue around the gemma periphery (Fig. 5 *D*–*F*) did not communicate the repressive apical dominance signal. Multiple regenerating meristematic regions emerged in the rest of the gemma, despite there being a fully functioning apical notch present. If the region around the original apical notch included significant amounts of surrounding tissue (Fig. 5 *E* and *F*), then the apical notch continued to grow and develop as normal (blue arrows), even as meristem regeneration occurred elsewhere (dashed circles).

If this region contained just the apical notch and cells immediately surrounding the notch, then the outcome was different (Fig. 5 *G* and *H*). The rest of the gemma was released from apical dominance and meristem regeneration proceeded (dashed circles). The original notch continued meristematic activity for the first day after ablation, indicated by enhancer trap marker signal persisting. At 2DaA this signal faded and cell division ceased, and by 3DaA the notch was inactive (gray arrows). This occurred in cases where the apical notch and immediately surrounding region was totally disconnected from the rest of the gemma (*SI Appendix,* Fig. S8).

This observed self-inhibitory feedback is characteristic of auxin (36, 54, 60). A reporter line marking activity of the canonical polar auxin transporter Mp*PIN1* promoter (37, 44) shows that in 0dpg gemmae signal is focused around the apical notches, is present around the gemma edge and in occasional cells across the gemma center. This supports findings that the apical notch is an auxin source (54) and suggests that auxin transport and signaling is more widespread. Marker line gemmae were ablated according to the incision patterns described above (Fig. 5 *C*, *F*, and *I*). Fig. 5*C* showed no expression central tissue connection, yet the intact notch retained apical dominance over the rest of the gemma. Conversely, Fig. 5 *F* and *I* show that peripheral connections had Mp*PIN1* promoter activity, yet meristem regeneration occurred elsewhere in the gemma (dashed circles). The reduced Mp*PIN1* promoter signal around the apical notch by 3DaA in Fig. 5*I* reflected the loss of meristematic activity in that notch (gray arrow). This also occurred in Mp*YUC2* marker line gemmae (Fig. 5*J*), where there was loss of Mp*YUC2* promoter activity in the isolated notch as it ceased proliferation and the cells expanded. Elsewhere there was widespread Mp*YUC2* promoter activity at 1DaA, indicating reorganization of auxin biosynthesis activity due to falling auxin levels in the rest of the gemma. Mp*YUC2* promoter activity then became progressively more focused to smaller areas as meristem regeneration proceeded.

### Larger Gemma Fragments Regenerate Meristems Faster Than Smaller Gemma Fragments.

**Fig. 6.**
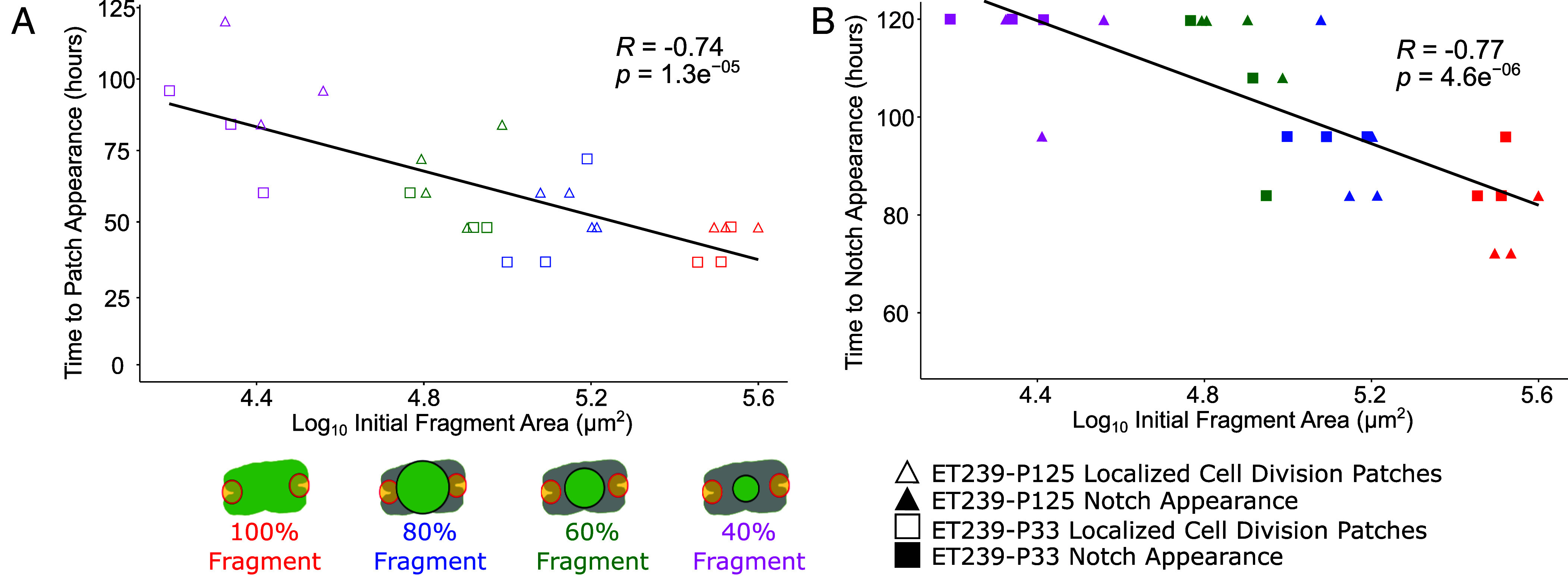
A 108-h time course using two different enhancer trap apical notch/meristem marker lines shows that larger gemma fragments have faster emergence of patches of localized cell division and faster appearance of morphologically recognizable notches. Plotting log_10_ fragment area versus time to appearance of localized patches of cell division (*A*) or notch morphology (*B*) finds a significant Pearson’s correlation coefficient between fragment size and regeneration speed. Where patches or notches had not appeared within the duration of the experiment, time was given as 120 h. A schematic of the ablation pattern used is given at the bottom, with orange bounded by red indicating an entirely excised apical notch. The black circle shows the laser ablation circle trace used, with the circle diameter calculated as a percentage of the original notch–notch distance. Open symbols denote appearance of patches of localized cell division with enhancer trap marker signal; closed symbols denote when notch morphology within a dense patch of marker signal was first observed; symbol colors relate to the percentage fragment size as indicated in the schematics. See *SI Appendix,* Fig. S9 for regeneration timings and *SI Appendix*, Fig. S10 for a representative set of time course images.

Since the size of the surrounding region has an effect on apical notch activity, I examined how the size of the gemma fragment influences meristem regeneration. A standard-sized area around the apical notch region was entirely excised in a number of gemmae from the enhancer trap lines ET239-P125 and ET239-P33. The remaining gemma fragments were then ablated to leave a circle of tissue with a diameter 40, 60, 80, or 100% the notch-to-notch distance of the original gemma. The reappearance of the enhancer trap marker signal and the appearance of notch morphology was used to measure the speed of meristem regeneration (Fig. 6 and see *SI Appendix,* Fig. S9 for regeneration timings and *SI Appendix,* Fig. S10 for a representative set of images from the time course). The timings of when patches of localized cell division with marker signal appeared (dashed circles, *SI Appendix,* Fig. S10), and when notch-type morphology re-formed (yellow arrows, *SI Appendix,* Fig. S10) were recorded (Fig. 6 and *SI Appendix,* Fig. S9). Over the 108-h time course it was observed that larger fragments had patches of marker signal reappear significantly earlier (Fig. 6*A*) and had significantly faster regeneration of apical notch morphology (Fig. 6*B*) compared to smaller fragments.

## Discussion

### Laser Ablation of Transgenic Marchantia Lines Reveals Minimum Requirements for Meristem Regeneration.

**Fig. 7.**
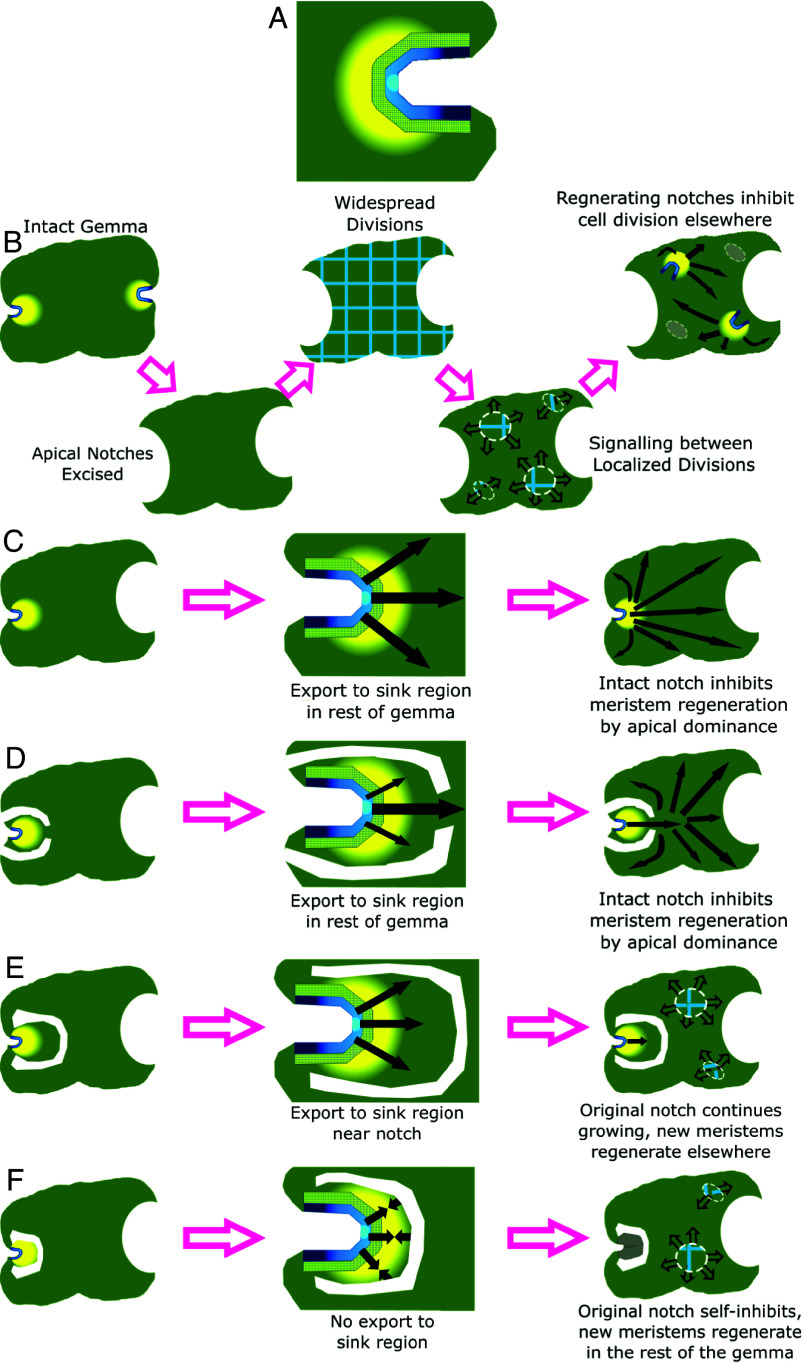
A model of apical notch organization, meristem regeneration, and apical dominance signaling in Marchantia. (*A*) The stem cells in the first row of the apical notch (in blue) are critical for (pre)meristem maintenance. The cells at the center of this population are distinct from the cells toward the edge (blue gradient). The cells in the two rows behind are primed for meristem regeneration (blue hatching). The stem cells emit a signal regulating cell division and expansion in the surrounding region (yellow gradient). (*B*) Process of meristem regeneration after excision of both apical notches. Initially there are widespread cell divisions (blue grid), then cell divisions are restricted to localized patches (circles) which communicate (open arrows). Eventually one or more of these patches passes a developmental threshold and produces a repressive apical dominance auxin signal (black arrows) that inhibits cell division elsewhere. (*C*) When one apical notch is intact, it emits an apical dominance auxin signal (black arrows) that prevents the emergence of any new meristems. (*D*) When one apical notch is intact and linked to the rest of the gemma fragment through a central connection, then apical dominance signal is exported (black arrows) and no new meristems regenerate. (*E* and *F*) When one apical notch is intact and linked to the rest of the gemma fragment through a peripheral connection, no apical dominance signal is exported (black arrows), and meristem regeneration occurs elsewhere in the gemma fragment (circles). If the intact apical notch is surrounded by sufficient auxin sink tissue then it continues meristematic activity (*E*). If the intact apical notch is not surrounded by sufficient sink tissue, then auxin builds up and this notch becomes inactive (marked in gray) due to self-inhibition (*F*).

Previous experiments investigating Marchantia regeneration were limited to surgical manipulations on older thalli, removing all of the apical notch with scalpel blades (8, 11, 12, 27). These cutting experiments demonstrated that only when all apical notches were removed from a thallus would new meristematic regions emerge. Laser ablation microscopy allows for finer scale disruptions of the apical notch premeristem of 0dpg gemmae, which is more accessible for ablation than the fully mature meristems of older gemmae. Laser ablation demonstrated that removal of the first row of cells in the apical notch is sufficient to induce meristem regeneration. It reveals a previously unappreciated substructure within the premeristem of the 0dpg gemma (Fig. 7*A*), with the immediate first row of apical notch stem cells being critical for (pre)meristem maintenance and the second and third rows being “primed” for meristem regeneration in preference to the rest of the gemma. The cells in these rows are divisionally active and have the Mp*ARF2*-related gene expression networks of early ontogeny (54), therefore less reprogramming would be involved to acquire meristem identity compared to other cells.

Beyond this there appears to be no wider predictability as to which regions will regenerate new meristems (Fig. 7*B*). The exception is the stalk scar region, which was never observed to regenerate new meristem. These are the oldest cells in the gemma, produced at the initiation of gemma formation within the gemma cup (18, 22). It may be the case that, just as the youngest cells in the gemma are primed for meristem regeneration, the oldest cells are excessively complex to reprogram. This regenerative flexibility was reported in early studies (12) and is in contrast to vascular plants, where in the postembryonic plant only certain somatic cell types can form new meristem. It also differs from cutting experiments done on older thalli, where regeneration always occurred at the apical midrib on the basal explant (8, 11).

### Apical Notch Organization and Communication.

The standard model of the Marchantia meristem involves a single pluripotent apical cell with four division planes, located at the center (apex) of the notch. This apical cell divides to form dorsal, ventral, and lateral merophytes/subapical cells, which then go on to form the various tissues of the thallus (16, 18, 19). The apical cell in the 0dpg gemma premeristem has primarily been defined by morphological characterization rather than direct experimental evidence (18, 24, 46). For dichotomous branching and apical notch bifurcation, two apical cells must be present (45, 46). There is debate over the bifurcation mechanism: de novo formation of a new apical cell at a distance from the original one; loss-of-fate of the original apical cell and de novo formation of two new apical cells some distance from each other; or division of the original apical cell to produce two new apical cells, which each then divide, producing subapical cells between them and displacing the two apical notches apart. In more complex plant meristems (e.g., angiosperm SAM) there is a population of pluripotent cells that divide and maintain the meristematic stem cell niche (32).

I tested how this model applies to the premeristem of the 0dpg apical notch experimentally, by ablating portions of the crucial first cell row of the apical notch (i.e., the apical cell and subapical cells). This showed that the important factor is the number of intact cells in this row that are in contiguous contact. The notch apex (containing the apical cell) seemingly has a greater functional capacity than the cells on the sides of the notch, since this central subpopulation alone could sustain meristematic activity if no other apical notches were present in the gemma. This confirms communication between apical notches within a gemma.

When the first row of apical notch cells was ablated to remove one side and part of the notch center, there was a sufficiently large contiguous subpopulation of cells to sustain cell proliferation and apical dominance (Fig. 3). After 4 d the apical notch had reorientated itself, with a new apex where the center of the intact contiguous subpopulation of cells was originally. Apical notch mechanistic processes also shifted, shown by the focusing of Mp*YUC2* promoter activity around the reorientated notch apex (Fig. 3*C*). This was not de novo meristem regeneration of a new apical notch; instead it appears to be a case of remodeling, where divisions of the subapical cells create a new apex, presumably with an apical cell at its center. This happens even under conditions where meristem regeneration cannot occur, for example if another apical notch is present in the gemma, or in the presence of 1-NAA, an inhibitor of meristem regeneration. That apical notch reorientation (Fig. 3) is not de novo meristem regeneration is supported by the fact that no new patches of cell division emerge elsewhere in the gemma (cf Fig. 4). It also suggests that the reorientation process is to some extent decoupled from auxin signaling, unlike de novo meristem regeneration.

These experiments demonstrate that there is a population of stem cells in the apical notch. What is important for cell proliferation and apical dominance is that there is a sufficient number or “quorum” of these cells intact and in contiguous contact. The characteristic notch morphology emerges from differential rates of cell division and expansion from the quorum center to the edges (61). The new apex of the reorientated notch is at the center of this quorum, not necessarily the cells that were closest to the original notch apex and apical cell. Under this updated model, the “Apical Cell” is not a permanent cell identity that is defined at gemma germination but instead emerges from the communication dynamics within the notch. It is this feedback process (45, 46, 49) across the entire stem cell population that sustains meristematic activity.

The requirement for contiguous contact means that cells within the apical notch must communicate directly. The importance of such paracrine signaling in Marchantia thalli has been noted previously, where disrupting plasmodesmata connections caused cells to independently regenerate new thalli (62). Paracrine signaling would provide a mechanism to regulate cell division rates from the apex to the sides of the notch, and offer a basis for dichotomous branching, as previously suggested by Hirakawa et al. (45, 46). The formation of two distinct, active meristematic centers could be closely regulated if they arise from one contiguous population of apical notch cells. These two apical notch meristems could coexist in close proximity because each consists of an equally sized quorum. A quorum of dividing cells may also be required for the production of apical cells, for example in the sporeling protonema-to-prothallus transition (9, 20, 55).

### Partial Apical Notch Ablation Reveals That Meristem Regeneration Is a Flexible Process.

Multiple regions in a gemma can begin the regeneration process, but not all will eventually form apical notches (9), just as not all apical notches are proliferative (21, 26, 27). This is attributed to some regenerating regions having apical dominance or lateral inhibition effects (11). Ablation experiments (Fig. 4 and *SI Appendix,* Fig. S6) suggest that there are certain developmental stage thresholds that govern dominance during regeneration. The partially ablated notch reasserted apical dominance over larger patches of cell division, showing that developmental stage is more important than the size of the regenerating region.

Loss of meristematic activity can be reversed, and de novo meristem regeneration can be stopped even at a late stage. There may be a hierarchy of threshold stages during the processes of meristem formation and regeneration. This would explain the strong apical dominance effects of the apical notch premeristems in the germinating gemma, even before full meristem maturity. Since the partially ablated notch in Fig. 4 was already composed of stem cells, it progressed faster to the key threshold stage for apical dominance. Regenerating regions that have passed specific thresholds could dominate other, less mature regions, while multiple regions could coexist if they develop simultaneously (8, 9, 45, 46). This could provide a checkpoint mechanism for meristem regeneration and prevent too many regions emerging in too close proximity, and explain how bifurcating meristems could coexist in close proximity, if they coordinate development through threshold stages or are beyond a threshold stage at formation.

### Apical Dominance Signal Transport across the Gemma.

The partial notch ablation experiment shows that apical dominance signaling in Marchantia is an active process that can be temporarily disrupted and reestablished. Lasering incisions demonstrates that apical notches produce a signal that flows outward to influence the rest of the gemma (Fig. 7*C*). Incisions parallel to the notch (*SI Appendix,* Fig. S7 *F*–*O*) show more cell division proximal and more cell expansion distal to the incision. This was only observed when the portion of the notch closest to the incision was intact, irrespective of the integrity of the cells at the notch apex. Cells distal to the incision did not begin meristem regeneration, so while some signals flow directly from the notch, the apical dominance signal was transported around the parallel incisions.

Further experiments to test which regions conduct the apical dominance signal found a difference between connections formed from central and peripheral tissue (Figs. 5 and 7 *D* and *E*). This was not dependent on connection width, as central connections 1-2 cells wide did not see meristem regeneration, but wider peripheral connections did. This means that the apical dominance signal must be carried by a specific mechanism, and not all cells possess this mechanism. This may link to the observation that most auxin transport and meristem regeneration occurs along the ventral midrib of mature thalli (8, 11, 63), though gemmae have no midribs or defined dorsoventrality at germination (18, 24).

Auxin is the prime candidate for this apical dominance signal. Auxin regulates processes such as apical dominance (6) and meristem regeneration (7) in all plants, including Marchantia (28, 29). A reporter line marking promoter activity of the auxin biosynthesis gene Mp*YUC2* (26, 64) shows that at 0dpg there is signal at the apical notches and throughout the gemma, but this becomes more focused around the notch in older gemmae (9). This shows that auxin is produced in the apical notch and must be exported to the rest of the thallus, confirming recent findings by Flores-Sandoval et al. (54).

That study suggested that the canonical MpPIN1 is responsible for auxin transport immediately surrounding the apical notch but did not examine longer range auxin signaling across the gemma. Canonical PIN distribution (9, 37, 44) does not explain why an auxin-based apical dominance signal is exported through central connections but not peripheral connections. Mp*PIN1* promoter expression is high at the gemma edge, present in peripheral connections and absent from central connection cells (Fig. 5 *C* and *F*). Furthermore, meristem regeneration still occurs in Mp*pin1* mutants, albeit more slowly (53). It may be the case that other auxin transporters (35, 37, 65) are involved. The Marchantia noncanonical short PINs are promising candidates as recent work found that they localize to the plasma membrane and are capable of auxin export. MpPINW has an asymmetric distribution in the plasma membrane, and this asymmetry appears to point centrally rather than peripherally in cells around the apical notch (66). It is notable that meristem regeneration involves widespread Mp*PIN1* and Mp*YUC2* promoter activity, subsequently followed by marker signal concentrating around those regions of localized cell division. This means that meristem regeneration involves reorganization of the auxin transport machinery as well as auxin biosynthesis (dashed circles, Fig. 5 *F*, *I*, and *J*) (53).

### Balance between Auxin Sources and Sinks Is Required for Marchantia Meristem Maintenance and Regeneration.

Auxin has a self-inhibitory effect on its own biosynthesis (36, 60) and elevated auxin levels prevent meristem regeneration (8, 25). In Marchantia, this self-inhibitory effect is believed to be mediated by auxin up-regulating Mp*ARF1* expression, which then antagonizes *MpARF2* expression and auxin synthesis (54). This means there must be balanced auxin transfer between sources, where auxin is produced, and sinks, where auxin is degraded (26, 64, 67). The MpR2DII degron reporter system has shown that the apical notch meristem is the auxin source while the surrounding tissue acts as an auxin sink (54).

If the auxin sink is too large compared to the source then apical dominance is lifted and new meristems emerge; if the sink is too small compared to the source then auxin build-up interferes with meristematic activity. In Marchantia, an example of the former is seen in the short-term response to partial apical notch ablation (Fig. 4). Examples of the latter are apical notches isolated by peripheral connections (Fig. 5 *D*–*I*), or by complete separation from the rest of the gemma fragment (*SI Appendix,* Fig. S8). Here, the cells immediately surrounding the apical notch are the only available sinks. When this region is large the isolated notch continues growing normally (Fig. 7*E*), but when this is small the sink is insufficient, accumulating auxin self-inhibits and the apical notch ceases cell proliferation (Fig. 7*F*).

The auxin source-sink balance is also disturbed by apical notch removal. With no sources, auxin levels fall across the remaining fragment. Subsequently new sources (regions of cell division) emerge (Fig. 5*J*), until the balance is restored by the regeneration of apical notches (8). A hypothesis that emerges from this is that if there are a greater pool of cells to act as auxin sources or sinks, then regeneration will proceed faster compared to fragments with fewer cells. The results of the experiments presented in Fig. 6 (*SI Appendix,* Figs. S9 and S10) support this. An extreme version of the relationship between fragment size and regeneration speed is the case of isolated thallus cells (9, 10, 68). Here, it can take 5 to 7 d for cell divisions to occur, with recognizable notches only appearing after 14+ days. Marchantia meristem regeneration presents a simplified system to study auxin self-inhibition and the balance between auxin sources and sinks. This provides a basis for research into more complex scenarios, e.g., lateral organ formation (3, 6), wounding responses (57, 58).

## Conclusions

An outline model for the apical notch of a 0dpg Marchantia gemma is given in Fig. 7. A population of stem cells are found in the first row of the apical notch, behind which are two rows of cells primed to acquire stem cell identity. The center of the stem cell population forms the apex of the notch by having lower rates of cell division compared to the edges. The stem cells produce intranotch signals maintaining the quorum, short-range signals regulating cell division and expansion, and long-range signals sustaining apical dominance across the gemma. The apical dominance signal, likely auxin, is carried through the center of the gemma and not the peripheral regions. If neither apical notch has a sufficient quorum of intact stem cells in contiguous contact, or if the apical dominance signal cannot flow to the rest of the gemma, then new meristems emerge elsewhere in the gemma.

## Materials and Methods

Vector constructs were built according to the Loop Assembly protocol (41, 69) from L0 parts, L1 and L2 plasmids in the OpenPlant Loop Assembly toolkit (41). Lines used for each figure are given in *SI Appendix, Supporting Information Text*. Full plasmid sequences for vector constructs are given in Dataset S1. Plasmid vectors were transformed into Marchantia sporelings (Cam accession) by agrobacterium-mediated transformation according to previously described methods (41, 42). T0 sporelings and subsequent G1 plants were grown at 21 °C under continuous light (intensity = 150 μmol/m^2^/s) on 1.2% w/v agar (Melford capsules A20021) plates of Gamborg B5 media with vitamins (Duchefa Biochemie G0210) prepared at half the manufacturer’s recommended concentration and adjusted to pH 5.8. The growth medium was supplemented with 100 µg/mL Cefotaxime (BIC0111; Apollo Scientific, Bredbury, UK) to reduce bacterial contamination, 20 µg/mL Hygromycin (10687010; Invitrogen) for selection and 3 µM 1-NAA (N1641, Sigma-Aldrich) for regeneration inhibition experiments.

Imaging was carried out using a Leica Sp8 upright confocal microscope or Zeiss LSM800 upright confocal microscope. Laser ablation experiments were performed using a Leica LMD6000 laser dissection microscope system or Zeiss PALM MicroBeam Laser Capture Microdissection Microscope. Microscopy settings are given in *SI Appendix,* Table S1 and *Supplementary Information Text*. Images were processed using LasX, ZEN Blue, and Fiji/ImageJ software packages. Figures were produced using the QuickFigures (70) and Stitching (71) Fiji plugins. Consistent growth and marker signal responses were observed in at least three gemma replicates taken from two different lines, except for Fig. 4 and *SI Appendix,* Fig. S6, where reestablishment of apical dominance by a partially ablated apical notch was observed once. Pearson’s correlation coefficient analyses were performed using R Statistical Software v4.5.2 (The R Foundation for Statistical Computing).

Propidium iodide (PI) staining was used to verify that laser ablation did not cause widespread stress or damage to the gemma. WT and enhancer trap line gemmae were incubated in 10 µL of 100 µg/mL PI (Sigma) on a glass coverslip for 10 min at room temperature, ensuring that the gemma was not mechanically damaged or desiccated during incubation. Staining, ablation, and imaging were carried out in various orders to ensure that any PI signal relating to cellular damage was solely due to laser ablation. All experiments showed that the laser settings killed the targeted cells, cellular damage was localized to the ablated zone and there was no widespread cell death or stress (*SI Appendix,* Fig. S11). Thus, laser ablation microscopy is a suitable approach to study the microarchitecture of the apical notch region. The same concentrations and incubation times were used for PI staining of gemmae directly on the growth media at 0DaA, 4DaA, and 7DaA for *SI Appendix,* Fig. S5.

## Supplementary Material

Appendix 01 (PDF)

Dataset S01 (TXT)

## Data Availability

Study data are included in the article and/or supporting information.
