# Supplementary material for: Laser ablation microscopy reveals apical notch, apical dominance, and meristem regeneration dynamics in Marchantia polymorpha
Source: Proc Natl Acad Sci U S A. 2026 Jun 22;123(26):e2600460123. doi: 10.1073/pnas.2600460123 (PMC13320805; doi:10.1073/pnas.2600460123)
Supplement: Supplementary file 1 — Appendix 01 (PDF) [file pnas.2600460123.sapp.pdf]

PNAS

Supporting Information for

Laser ablation microscopy reveals apical notch, apical dominance, and meristem regeneration dynamics in Marchantia polymorpha.

Alan O. Marron[1,2*]

[1] Department of Plant Sciences, University of Cambridge, Downing Street, Cambridge CB2 3EA, United Kingdom
[2] Present Address: Oxford Brookes Centre for BioImaging, Oxford Brookes University, Headington Campus, Oxford OX3 0BP, United Kingdom

Email: amarron@brookes.ac.uk

This PDF file includes:

Supporting text
Figures S1 to S11
Table S1
Legends for Dataset S1

Other supporting materials for this manuscript include the following:

Dataset S1

Supporting Information Text

Transgenic plant lines used for each figure. ET numbers (ET239-PXXX) refer to the Marchantia enhancer trap screening project (1)

Fig. 1 D: L2_239-CsA (ET239-P64) F: L2_239-CsA (ET239-P153)
Fig. 2 L2_239-CsA (A: ET239-P21 B: ET239-P21 C: ET239-P153)
Fig. 3 L2_239-CsA (A: ET239-P21 B: ET239-P21) C: L2_283-CsA
Fig. 4 L2_239-CsA (ET239-P125)
Fig. 5 L2_239-CsA (B: ET239-P153 E: ET239-P153 H: ET239-P125) C, F, I: L2_268-CsA J: L2_283-CsA

Fig. S1 L2_239-CsA (A, B: ET239-P21 C,D: ET239-P33 E,F: ET239-P125 G,H: ET239-P153)
Fig. S2 L2_239-CsA (ET239-P153)
Fig. S4 L2_239-CsA (A:ET239-P125 B: ET239-P21 C: ET239-P21 D: ET239-P153 E: ET239-P21 F: ET239-P21)
Fig. S5 Wild Type (Cam accession)
Fig. S6 L2_239-CsA (ET239-P125)
Fig. S7 L2_239-CsA (B-E: ET239-P125 G-J, L-O: ET239-P153)
Fig. S8 L2_239-CsA (ET239-P21)
Fig. S10 L2_239-CsA (ET239-P125)
Fig. S11 A, B: Wild Type (Cam accession) C: L2_239-CsA (ET239-P14) D-H: L2_239-CsA (ET239-P64)

Microscopy Methods and Settings

Imaging was carried out using a Leica Sp8 upright confocal microscope and LasX v3.5.7.23225, equipped with a hybrid detector and a pulsed white-light laser, with a HC PL APO 10x/0.40 CS2 dry objective, a HC PL APO 20x/0.75 CS2 dry objective or a HC PL APO CS2 40x/1.30 oil objective. Images were taken at 1024x1024 frame size in photon counting mode with sequential acquisition, using bidirectional scanning at 600Hz and 2x line averaging. Time gating was active to supress autofluorescence. Imaging for SI Appendix Fig. S5 was done using a Zeiss LSM800 upright confocal microscope and ZEN Blue v2.6, with an EC Plan-Neofluar 10x/0.3 dry objective or a Plan-Apochromat 20x/0.8 M27 dry objective. SI Appendix Fig. S5 confocal images were taken at 1024x1024 frame size with a GaAsP-PMT detector with sequential frame acquisition, using bidirectional scanning at scan speed 8 and 2x averaging. Maximum-intensity projections of the images were obtained from z-stack series sliced at intervals between 1µm-12.5µm. Excitation and collection settings for each fluorophore are given in SI Appendix Supplementary Information Table 1.

Laser ablation experiments were performed using a Leica LMD6000 laser dissection microscope system equipped with a solid state 355nm cutting laser and controlled by Leica LMD6 software. Ablation performed under the 10x/0.3 HCX PL FL objective lens used settings 60 power, 45 aperture, 25 speed; ablation performed under the 40x/0.6 HCX PL FL objective lens used 60 power, 35 aperture, 20 speed. For SI Appendix Fig. S5 laser ablation experiments were performed using a Zeiss PALM MicroBeam Laser Capture Microdissection Microscope with 3 z-step cycles of 3µm; ablations performed under the 10x objective used settings 70 power, 90 focus, 10 speed; ablations performed under the 40x objective used 50 power, 25 focus, 10 speed. All gemmae used were taken directly from the gemma cup of the parent thallus (i.e., 0dpg). Intact gemmae were also taken from the same cups as controls to verify regular growth and marker expression. Gemmae were planted on 50mm agar plates and ablation and imaging performed directly on the plates to minimize mechanical disruption to the plants. For entire notch excision a 90µm diameter circle was drawn with its centre point at the notch apex, and all tissue within this circle was destroyed by laser ablation. All other cells or regions ablated are shown in schematics accompanying the relevant figures.

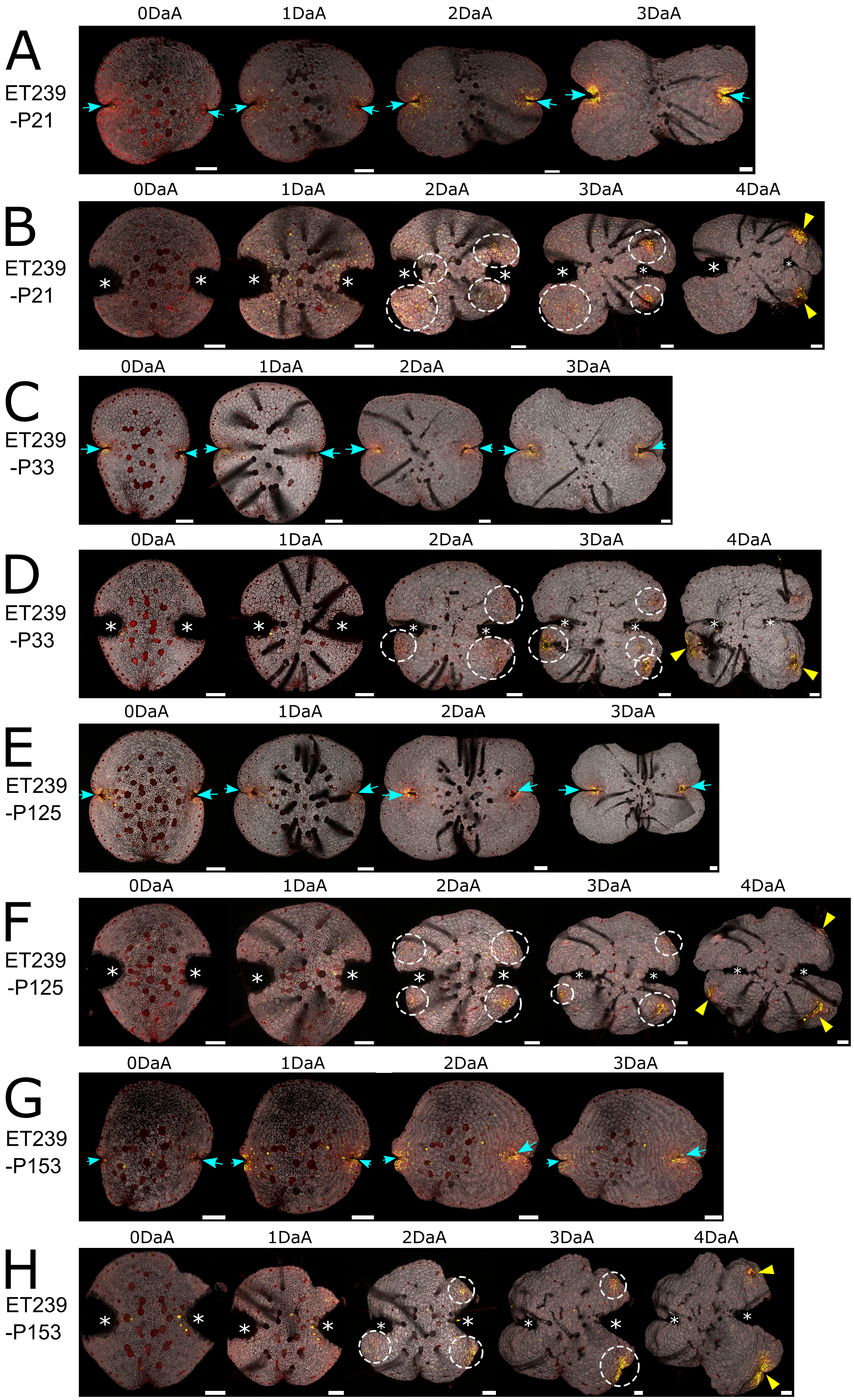
A
ET239
-P21
0DaA
1DaA
2DaA
3DaA
B
ET239
-P21
0DaA
1DaA
2DaA
3DaA
4DaA
C
ET239
-P33
0DaA
1DaA
2DaA
3DaA
D
ET239
-P33
0DaA
1DaA
2DaA
3DaA
4DaA
E
ET239
-P125
0DaA
1DaA
2DaA
3DaA
F
ET239
-P125
0DaA
1DaA
2DaA
3DaA
4DaA
G
ET239
-P153
0DaA
1DaA
2DaA
3DaA
H
ET239
-P153
0DaA
1DaA
2DaA
3DaA
4DaA

Fig. S1. Apical notches and meristem regeneration in the enhancer trap apical notch/meristem marker lines used in this study. (A) is an intact gemma from line ET239-P21 imaged daily from 0dpg until 3dpg. (B) is a gemma from line ET239-P21 with both notches entirely excised by laser ablation, imaged daily from 0DaA until 4DaA. (C) is an intact gemma from line ET239-P33 imaged daily from 0dpg until 3dpg. (D) is a gemma from line ET239-P33 with both notches entirely excised by laser ablation, imaged daily from 0DaA until 4DaA. (E) is an intact gemma from line ET239-P125 imaged daily from 0dpg until 3dpg. (F) is a gemma from line ET239-P125 with both notches entirely excised by laser ablation, imaged daily from 0DaA until 4DaA. (G) is an intact gemma from line ET239-P153 imaged daily from 0dpg until 3dpg. (H) is a gemma from line ET239-P153 with both notches entirely excised by laser ablation, imaged daily from 0DaA until 4DaA. In all lines, mVenus (in yellow) is strongly expressed in the dense cluster of cells in and around the intact and meristematically active apical notch, marked by blue arrows. Reappearance of densely clustered mVenus expression following apical notch excision occurs in all lines (marked by dashed circles) and indicates patches of localized cell division, with newly regenerated notches marked by yellow arrows. The genes whose enhancer elements have been trapped by these lines are unknown (1). All lines express a constitutive mScarlet cell membrane marker (in red) and chlorophyll autofluorescence is shown in grey. The ablation pattern used is as in Fig. 1C. Scale bars= 100µm.

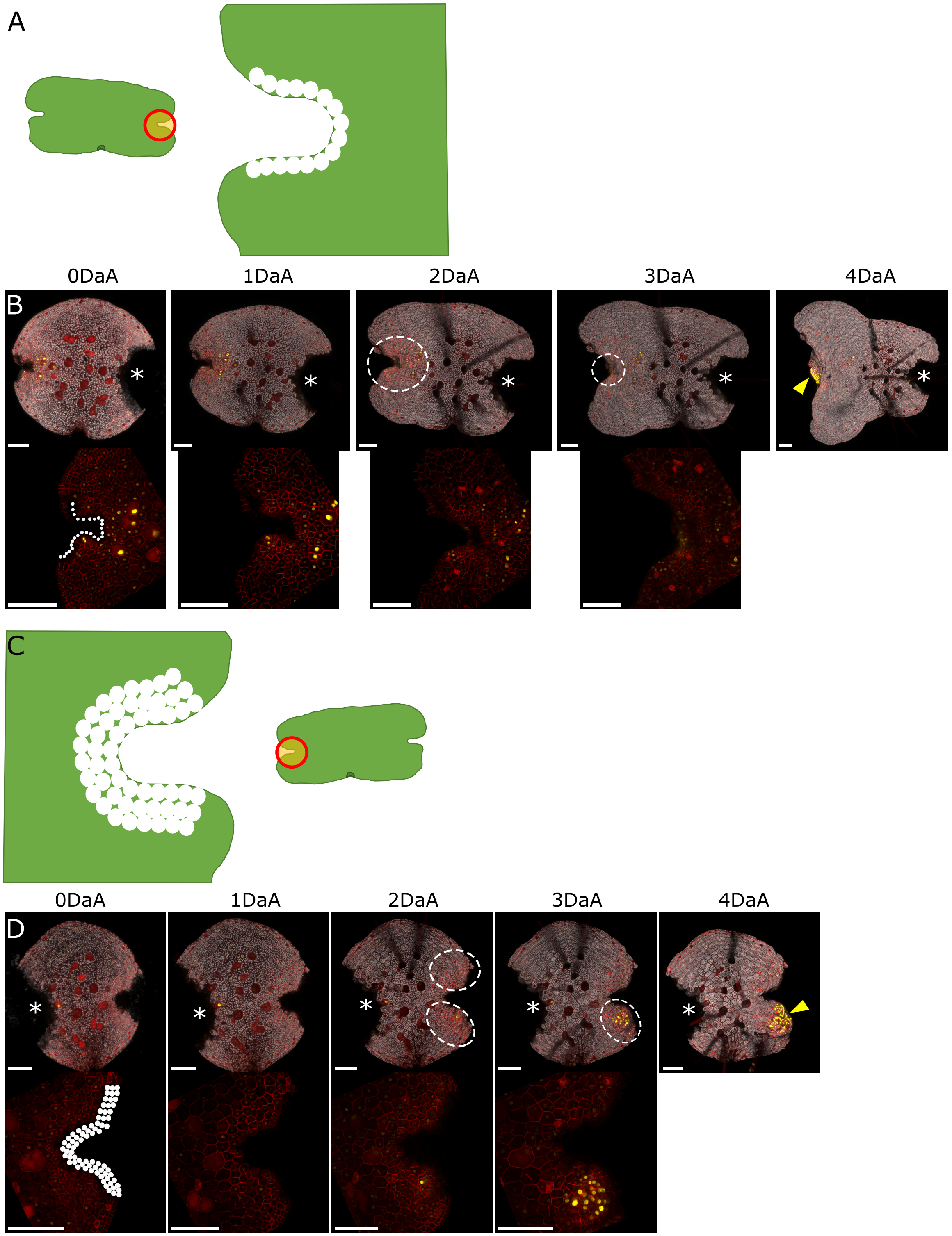

A
0DaA
1DaA
2DaA
3DaA
4DaA
B
C
0DaA
1DaA
2DaA
3DaA
4DaA
D

Fig. S2. Fine-scale ablation of the apical notch shows that the first three rows of cells are critical in determining meristem regeneration and its location. (A) and (C) are schematics for the ablation patterns used to ablate of the first row and first three rows of cells in the apical notch, respectively. White circles indicate ablated cells, orange bounded by red shows complete tissue excision. (B) and (D) are gemma from the enhancer trap apical notch/meristem marker line ET239-P153 imaged daily during a time course from 0DaA until 3DaA (notch close-up) or 4DaA (whole gemma). White circles mark the position of cells removed by laser ablation in the notch close-up images of 0DaA gemma. One notch in the gemma was entirely excised (marked by asterisk). (B) shows a gemma where only the first row of cells in the notch was ablated. This is sufficient to induce meristem regeneration by 4 DaA, but this occurs at the ablated notch, as seen by mVenus signal marking the localized patch of cell division (dashed circle) and regenerated notch (yellow arrow). (D) shows a gemma where the first three rows of cells in the notch were ablated. This was sufficient to allow patches of localized cell division (dashed circles) and eventually notch regeneration (yellow arrow) to occur elsewhere in the gemma, away from the original notch. Scale bars= 100μm.

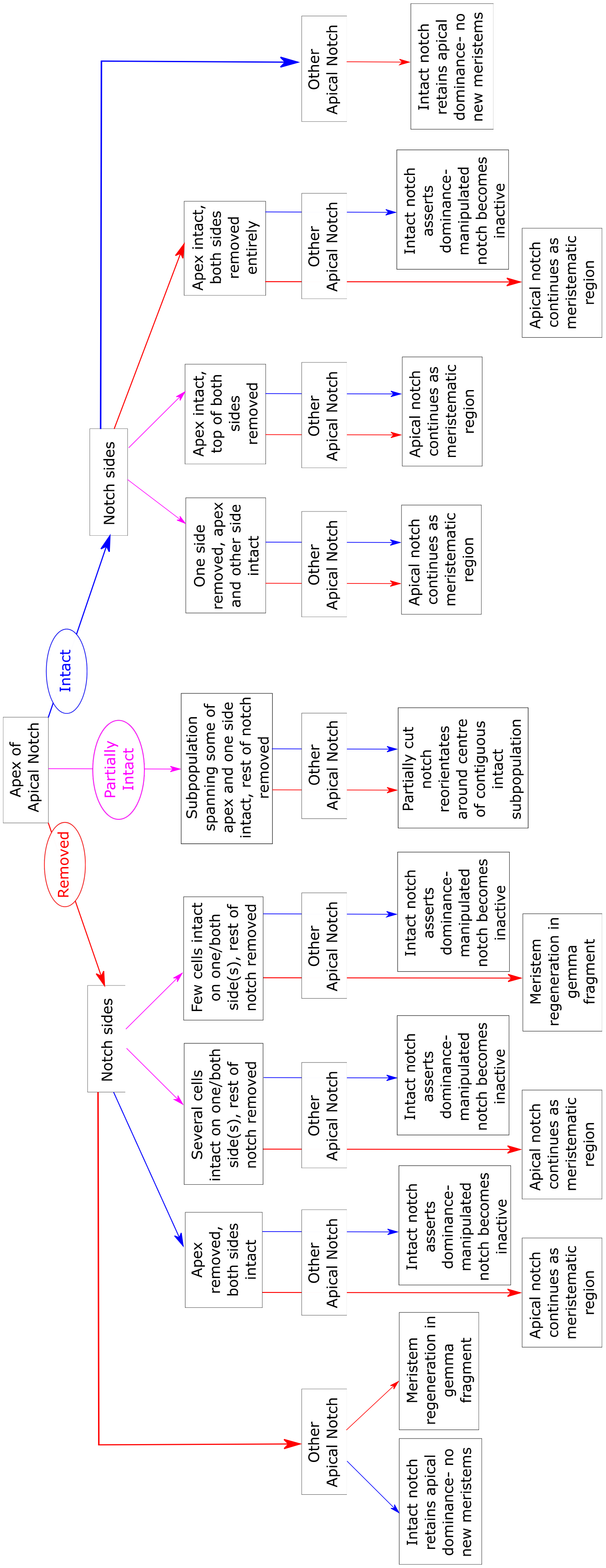

Apex of Apical Notch
Removed
Partially Intact
Intact
Notch sides
Notch sides
Other Apical Notch
Intact notch retains apical dominance- no new meristems
Meristem regeneration in gemma fragment
Apex removed, both sides intact
Several cells intact on one/both side(s), rest of notch removed
Few cells intact on one/both side(s), rest of notch removed
Other Apical Notch
Intact notch asserts dominance- manipulated notch becomes inactive
Apical notch continues as meristematic region
Meristem regeneration in gemma fragment
Subpopulation spanning some of apex and one side intact, rest of notch removed
Partially cut notch reorientates around centre of contiguous intact subpopulation
One side removed, apex and other side intact
Apex intact, top of both sides removed
Apex intact, both sides removed entirely

Fig. S3. Flow chart of outcomes of ablation experiments. The flow chart progresses from the status of the apex of the apical notch, through the status of notch sides and to the status of the other apical notch in the gemma. Red arrows indicate complete excision, magenta arrows indicate partial ablation, blue arrows indicate where a structure is left intact.

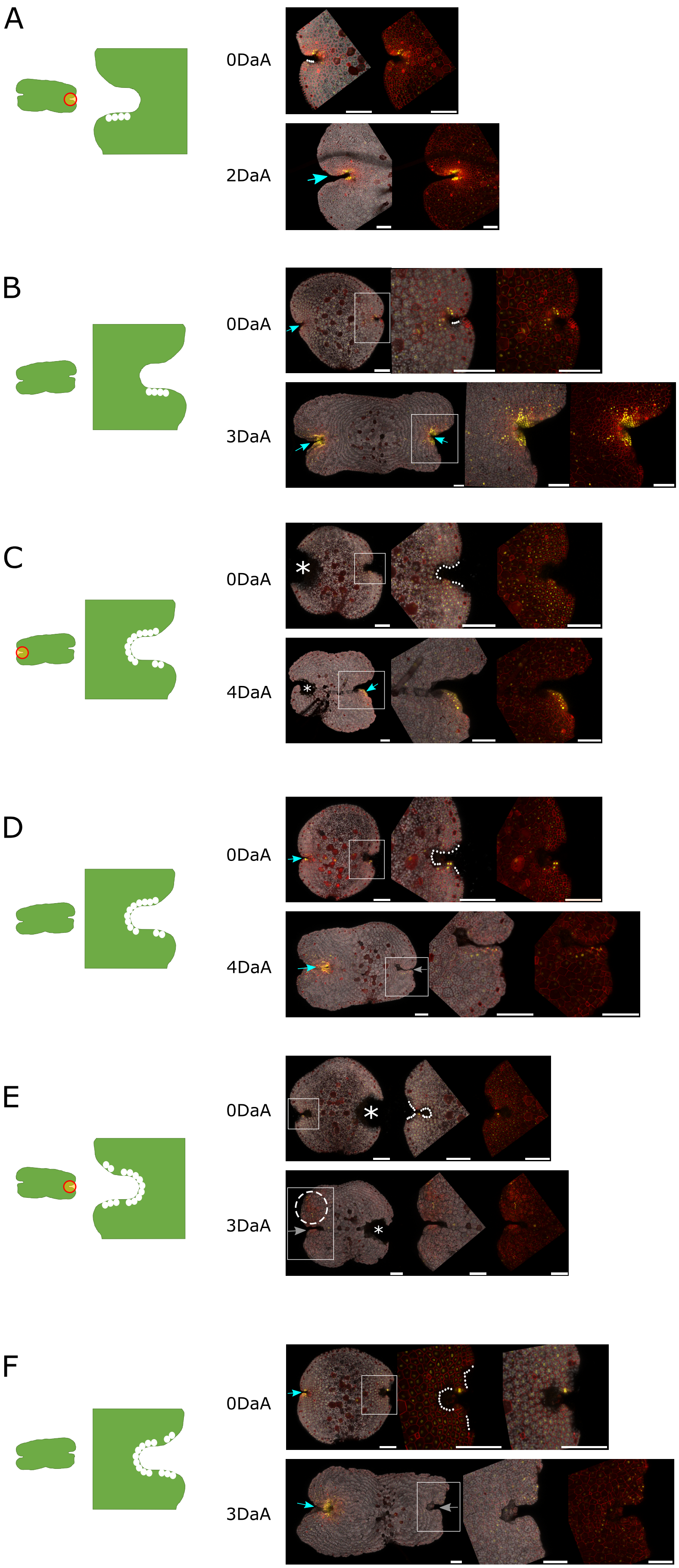
A
0DaA
2DaA
B
0DaA
3DaA
C
0DaA
4DaA
D
0DaA
4DaA
E
0DaA
3DaA
F
0DaA
3DaA

Fig. S4. Precise partial ablations of the apical notch. In each sub-figure a schematic of the ablation pattern is given (whole gemma and notch close-up), with white circles marking the location of ablated cells and orange bounded by red denoting excised tissue. Images of the 0DaA gemma are shown on the top row of each sub-figure (whole gemma, close-up of the apical notch area marked in the white box, same close-up without the chlorophyll autofluorescence channel) and the bottom row shows the same gemma at the indicated time point in the same format. Asterisks mark entirely excised notches, blue arrows mark active apical notches, grey arrows mark apical notches that have lost meristematic activity and dashed circles mark patches of localized cell division indicative of meristem regeneration. (A) and (B) demonstrate that ablating one side of the apical notch does not disrupt meristematic function irrespective of whether the other notch in the gemma is intact or not. Ablation of the notch, including the apex, leaving only a portion of one side intact does not disrupt meristem activity if the other notch is excised (C) but if the other notch is intact then meristematic activity ceases (D). Leaving only a few cells intact on both sides of the notch does not sustain meristematic activity, irrespective of whether the other notch is excised (E) or intact (F). If the other notch is excised then meristem regeneration occurs elsewhere in the gemma fragment (dashed circle, E). This is despite the total number of intact stem cells across the whole notch being greater than in the ablation experiment shown in (C). The enhancer trap lines used were ET239-P125 (A), ET239-P21 (B, C, E, F) and ET239-P153 (D). Scale bars= 100μm.

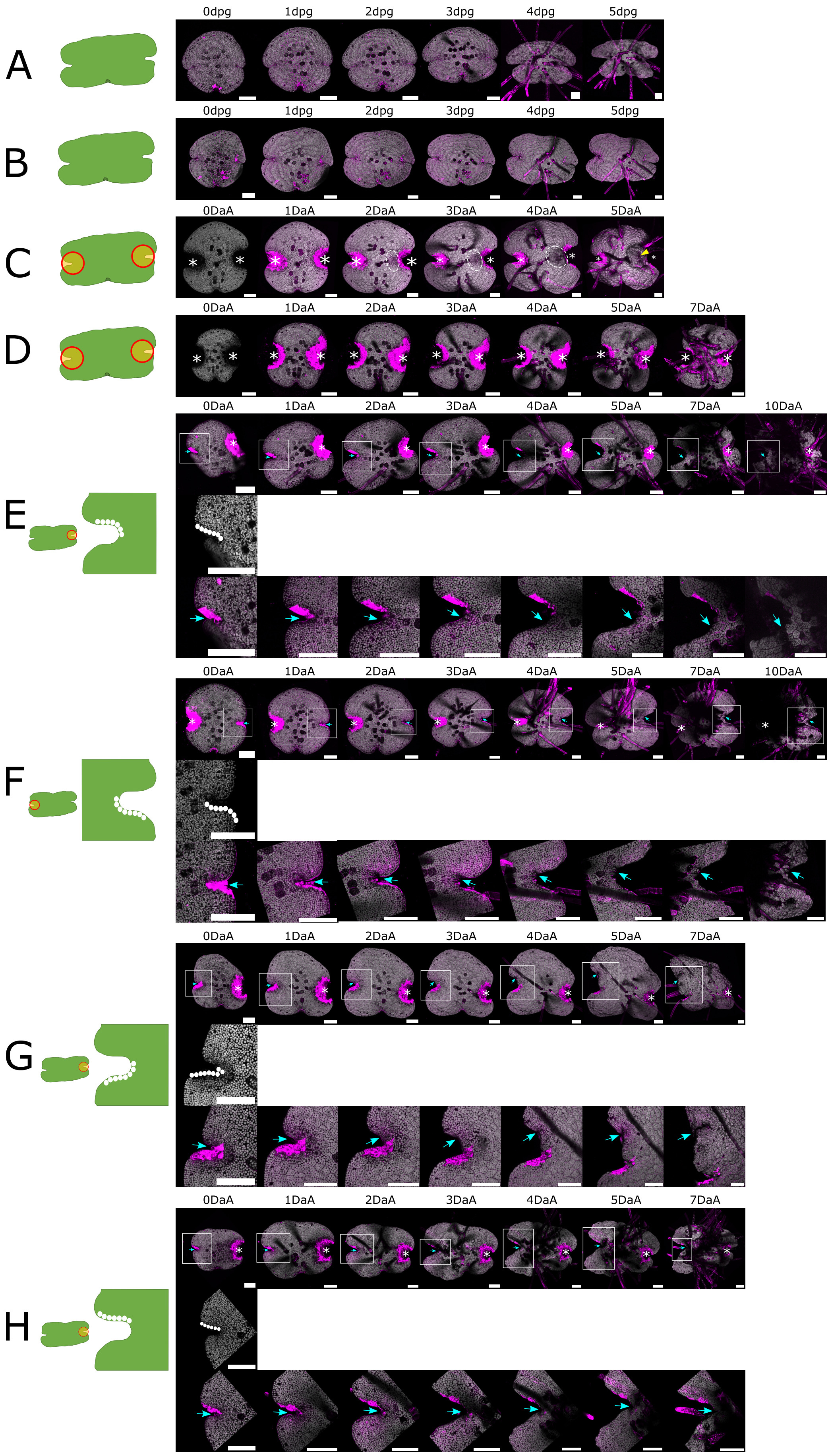

A
0dpg
1dpg
2dpg
3dpg
4dpg
5dpg
B
C
0DaA
1DaA
2DaA
3DaA
4DaA
5DaA
D
7DaA
E
10DaA
F
G
H

Fig. S5. Elevated auxin levels inhibit meristem regeneration but not apical notch reorientation. Gemmae grown on media supplemented with 3μM 1-NAA (A) overall had normal development during the first five days after germination (dpg), compared to gemmae grown on control media (B). The gemmae in (C) and (D) had their apical notches entirely excised (marked by asterisks) by laser ablation according to the schematic shown. While gemma fragments grown on control media (C) showed meristem regeneration (localized cell division=dashed circle, regenerated notch= yellow arrow), no meristem regeneration occurred on gemma fragments grown on media supplemented with 3μM 1-NAA (D). The time courses in (E) and (F) show that elevated auxin treatment did not inhibit apical notch reorientation. The notch changes shape, forming a new apex corresponding to the centre of the original intact subpopulation of stem cells, as in Fig. 3. This occurs at a similar speed to apical notch reorientation in gemmae grown on control media (G). The time course in (H) shows that if the centre of the apical notch is intact then no notch reorientation occurs, indicating that reorientation is not simply because of the generally altered notch morphology observed in gemmae grown in elevated auxin treatments. The schematic shows the ablation pattern used, with white circles marking ablated cells and orange bounded by red denoting excised tissue. Time courses shows the whole gemma, with an asterisk marking the entirely excised apical notch and a blue arrow indicating the position and orientation of the active apical notch. The white square indicates the area shown in the close-ups of the partially ablated apical notch region. All gemmae were wild-type Cam2 accession. Chlorophyll autofluorescence is shown in grey, propidium iodide staining in magenta. Scale bars= 100μm.

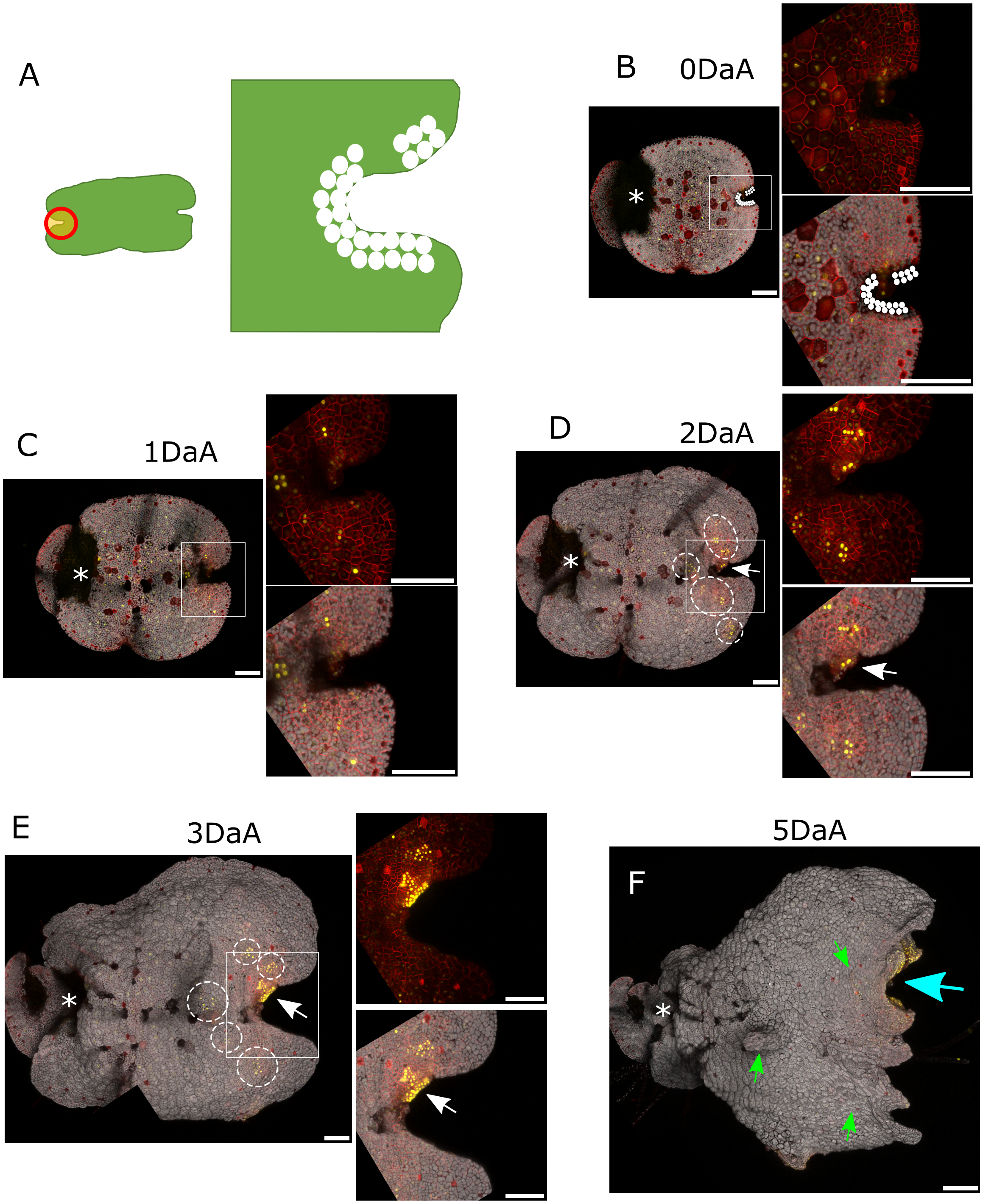
A
B
0DaA
C
1DaA
D
2DaA
E
3DaA
5DaA
F

Fig. S6. Re-establishment of apical dominance by a partially ablated apical notch. (A) shows a schematic of the ablation pattern used, with white circles marking ablated cells, orange bounded by red denoting excised tissue. (B)-(F) shows a time course of a gemma from the enhancer trap notch/meristem marker line ET239-P125. The images shown are of the whole gemma, together with close-ups of the ablated apical notch region in the white square (shown with and without the chlorophyll autofluorescence channel). Asterisks mark the entirely excised apical notch. (B) is the 0DaA gemma, with white circles marking ablated cells. (C) is the same gemma imaged at 1DaA, (D) at 2DaA, (E) at 3DaA. (F) is the whole gemma imaged at 5DaA. Although the original, partially ablated notch remained active (as indicated by the continuation of mVenus marker signal, white arrows), new patches of cell division emerged at 2DaA (D, dashed circles), with some persisting at 3DaA (E, dashed circles). However, by 5DaA (F) the original notch had reformed and re-established apical dominance (marked by blue arrow), as demonstrated by this notch being the only region of the gemma with mVenus marker signal. The areas that were patches of localized cell division stopped dividing (indicated by loss of mVenus signal) and remained as protrusions on the gemma surface (green arrows). These observations were recorded in one gemma replicate. Note that the image in E is missing a small portion at the bottom around where the stalk scar is located, due to incomplete coverage of the stitched fields of view. Scale bars= 100µm.

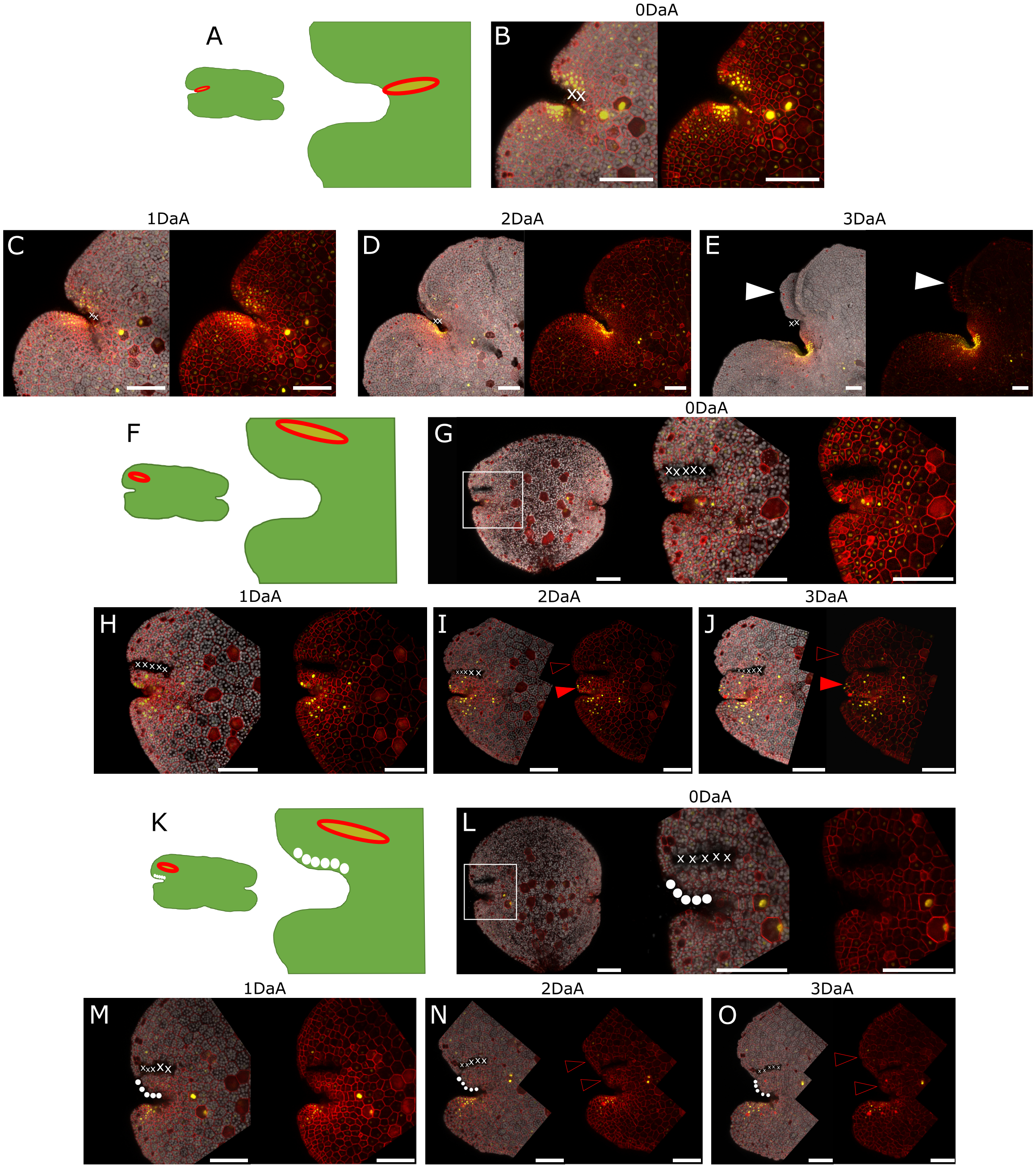
A
0DaA
B
1DaA
C
2DaA
D
3DaA
E
F
0DaA
G
1DaA
H
2DaA
I
3DaA
J
K
0DaA
L
1DaA
M
2DaA
N
3DaA
O

Fig. S7. Ablating an incision perpendicular to the apical notch has little effect, whereas ablating an incision parallel to the apical notch alters the balance between cell division and expansion either side of the incision site. (A) Schematic of the perpendicular ablation incision pattern, with orange bounded by red denoting excised tissue. (B) shows a close-up of the ablated notch, with white X marking ablated tissue. (C) is the same gemma imaged at 1DaA, (D) at 2DaA, (E) at 3 DaA. Ablation perpendicular to the apical notch had no obvious effect on notch function, other than the z-axis thallus split (1) in the gemma appearing earlier on the ablated side of the notch (white triangle), at 3DaA. (F) and (K) are schematics of the parallel ablation incision patterns, with orange bounded by red denoting excised tissue and white circles marking ablated cells. (G)-(J) are time courses from 0DaA until 3DaA of a gemma ablated in the patterns shown in (F), with white X symbols marking ablated tissue in (G). Proximal to the incision there was increased cell division (closed red triangle), whereas distal to the incision there was less cell division and more cell expansion (open red triangle), compared to the equivalent position on the non-ablated side of the notch. (L)- (O) is a daily time course from 0DaA until 3DaA of a gemma ablated as in (K), where in addition to the tissue ablated parallel to the apical notch (white X symbols in L) the apical notch cells directly proximal to the incision had also been ablated (white circles in L). In this case the cells on both sides of the incision showed reduced division and increased cell expansion (open red triangles) compared to the equivalent position on the non-ablated side of the apical notch. Gemma images are given in the format whole gemma (G and L only), notch close-up, notch close-up without the chlorophyll autofluorescence channel. Gemmae used were from the enhancer trap apical notch/meristem marker lines ET239-P125 for the perpendicular ablation experiment (B-E) and ET239-P153 for the parallel ablation experiments (G-J and L-O). Scale bars= 100µm.

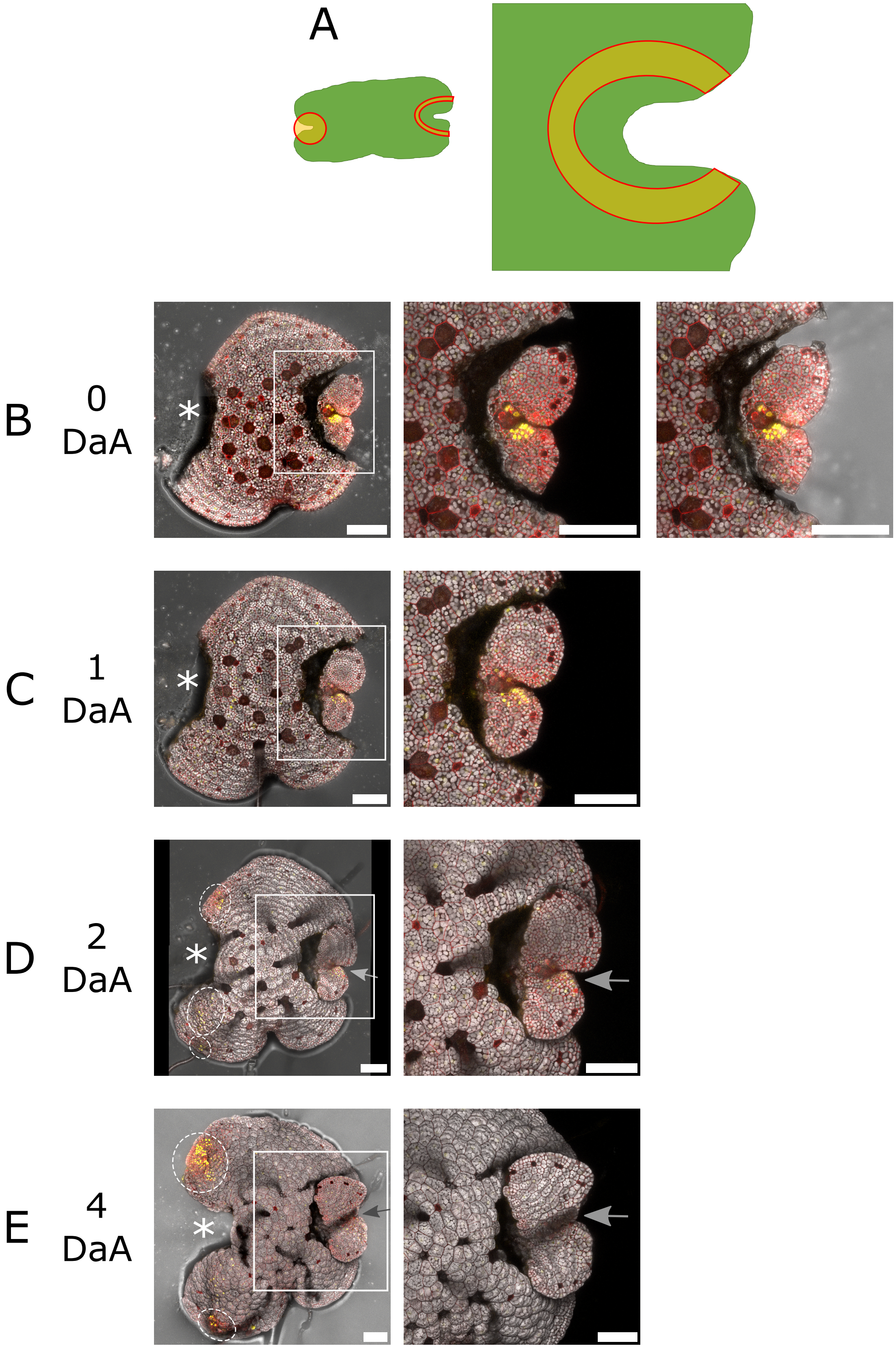
A
B
0
DaA
C
1
DaA
D
2
DaA
E
4
DaA

Fig. S8. Entirely ablating the tissue surrounding the apical notch results in the isolated notch losing meristematic activity. (A) Schematic of the ablation pattern used, where one notch was entirely excised and laser ablation was used to excise all tissue surrounding the other notch (excised tissue shown in orange bounded by red). The isolated apical notch itself was intact and surrounded by a small amount of tissue. The confocal images show a gemma from the enhancer trap apical notch/meristem marker line ET239-P21 with one apical notch isolated using this ablation pattern, imaged during a time course from 0DaA (B) through 1DaA (C), 2DaA (D) until 4DaA (E). The whole gemma image includes the transmitted light PMT channel (gain=315) in addition to the mVenus (yellow), mScarlet (red) and chlorophyll autofluorescence (grey) channels; the white box in the whole gemma image corresponds to the area shown in close-up. The 0DaA close-up includes the transmitted light channel image to confirm that the apical notch was fully isolated from the rest of the gemma fragment by destruction of the intermediate tissue. Appearance of dense regions of new mVenus signal (dashed circles) shows that this isolation removed the effects of apical dominance on the rest of the gemma and allowed patches of localized cell division to emerge. The isolated notch itself showed loss of mVenus signal, cell division ceased and the cells expanded, indicating that this region lost meristematic activity (grey arrows). Scale bars= 100µm.

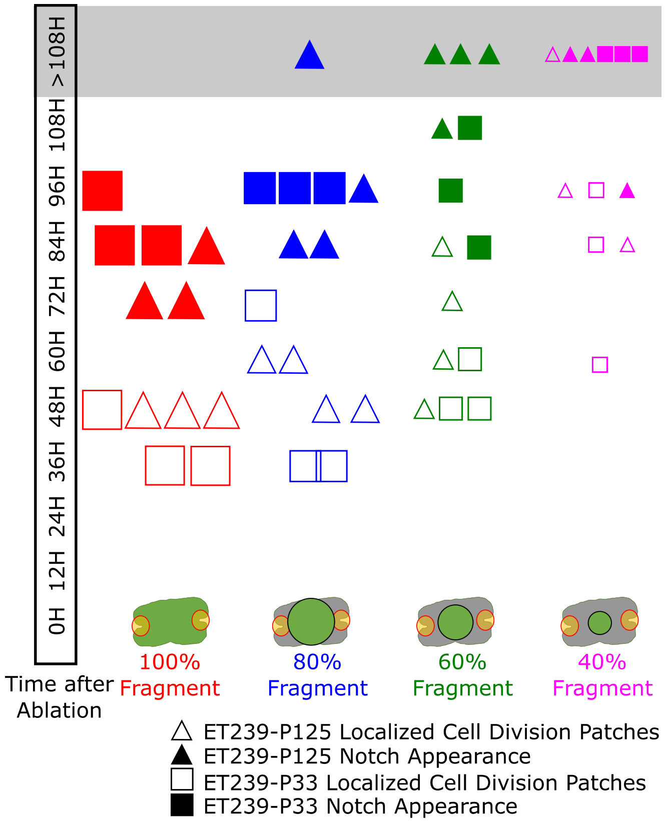

Fig. S9. A 108-hour time course using two different enhancer trap apical notch/meristem marker lines shows that larger gemma fragments have faster emergence of patches of localized cell division and faster appearance of morphologically recognizable notches. All 100% gemma fragments showed reappearance of notch morphology within the time course, as did all but one 80% fragment (shown by symbol in the grey >108H box). All 60% fragments had patches of localized cell division and marker signal emerge during the time course, but notches did not appear in all replicates. Only one of the 40% fragments had a notch reappear while one did not even form localized patches of cell division with marker signal. A schematic of the ablation pattern used is given at the bottom, with orange bounded by red indicating an entirely excised apical notch. The black circle shows the laser ablation circle trace used, with the circle diameter calculated as a percentage of the original notch-notch distance. Open symbols denote appearance of patches of localized cell division with enhancer trap marker signal; closed symbols denote when notch morphology within a dense patch of marker signal was first observed.

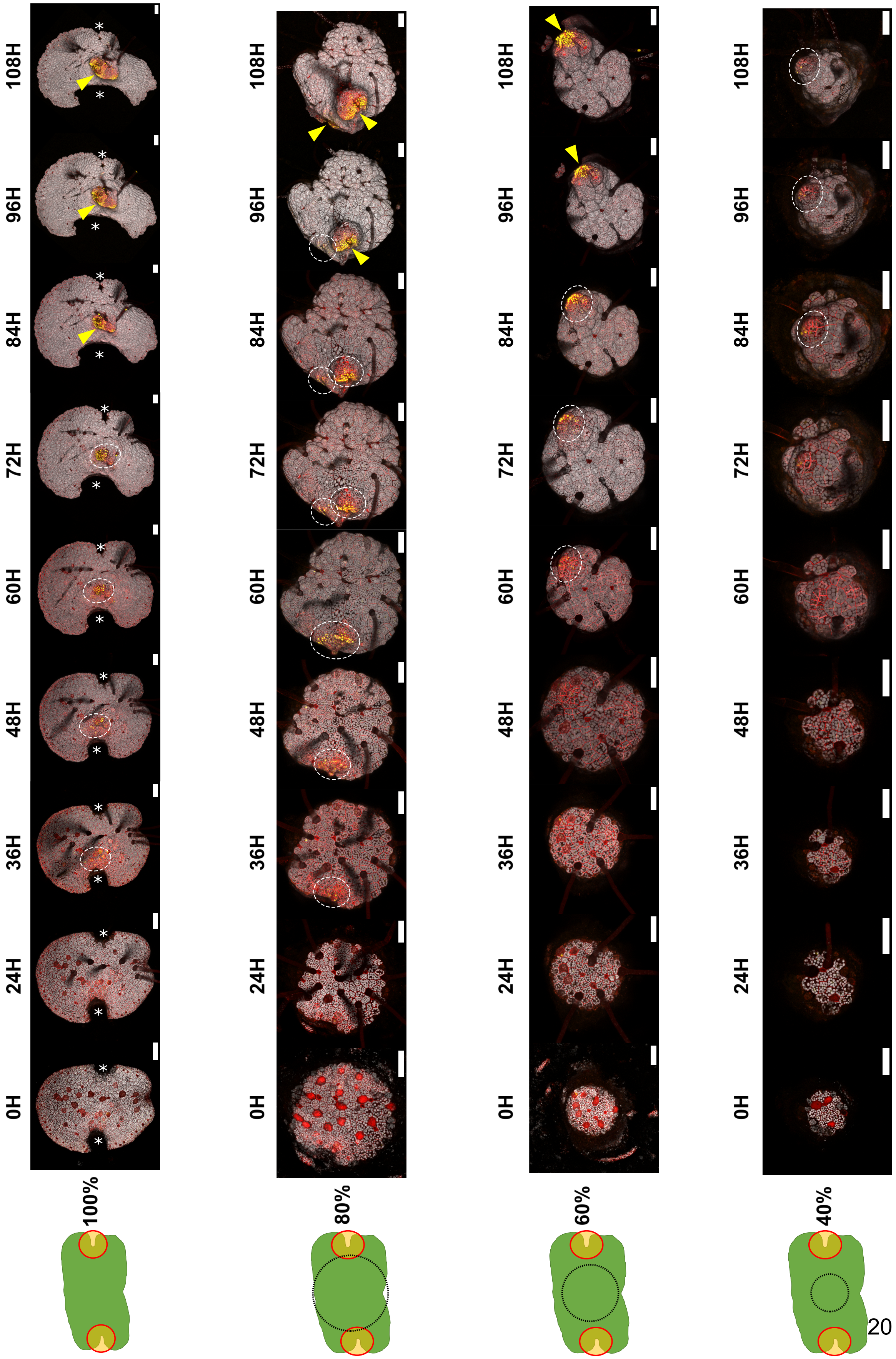
0H
24H
36H
48H
60H
72H
84H
96H
108H
100%
80%
60%
40%

Fig. S10. An example time course showing that meristem regeneration proceeds faster in larger gemma fragments. On the far left of each row is a schematic of the ablation pattern used, with orange bounded by red denoting excised tissue in the apical notch removal step (see Methods and SI Appendix Supplementary Information Text). The black circle shows the laser ablation circle trace used, with the circle diameter calculated as a percentage of the original notch-notch distance. Only the gemma fragment within the circle trace was retained, with all tissue outside of this destroyed. The emergence of localized patches of cell division, as defined by new, dense regions of mVenus marker signal, is indicated by dashed circles. The appearance of recognizable notch morphology within a region of dense mVenus marker signal is indicated by yellow arrows. These definitions were used to generate the data shown in Fig. 6 and SI Appendix Fig. S9. Both the emergence of localized patches of cell division and notch morphology occurred faster in larger fragments versus smaller fragments. Gemmae shown were from the enhancer trap apical notch/meristem marker line ET239-P125. Time indicated is in hours after ablation. Scale bars= 100μm.

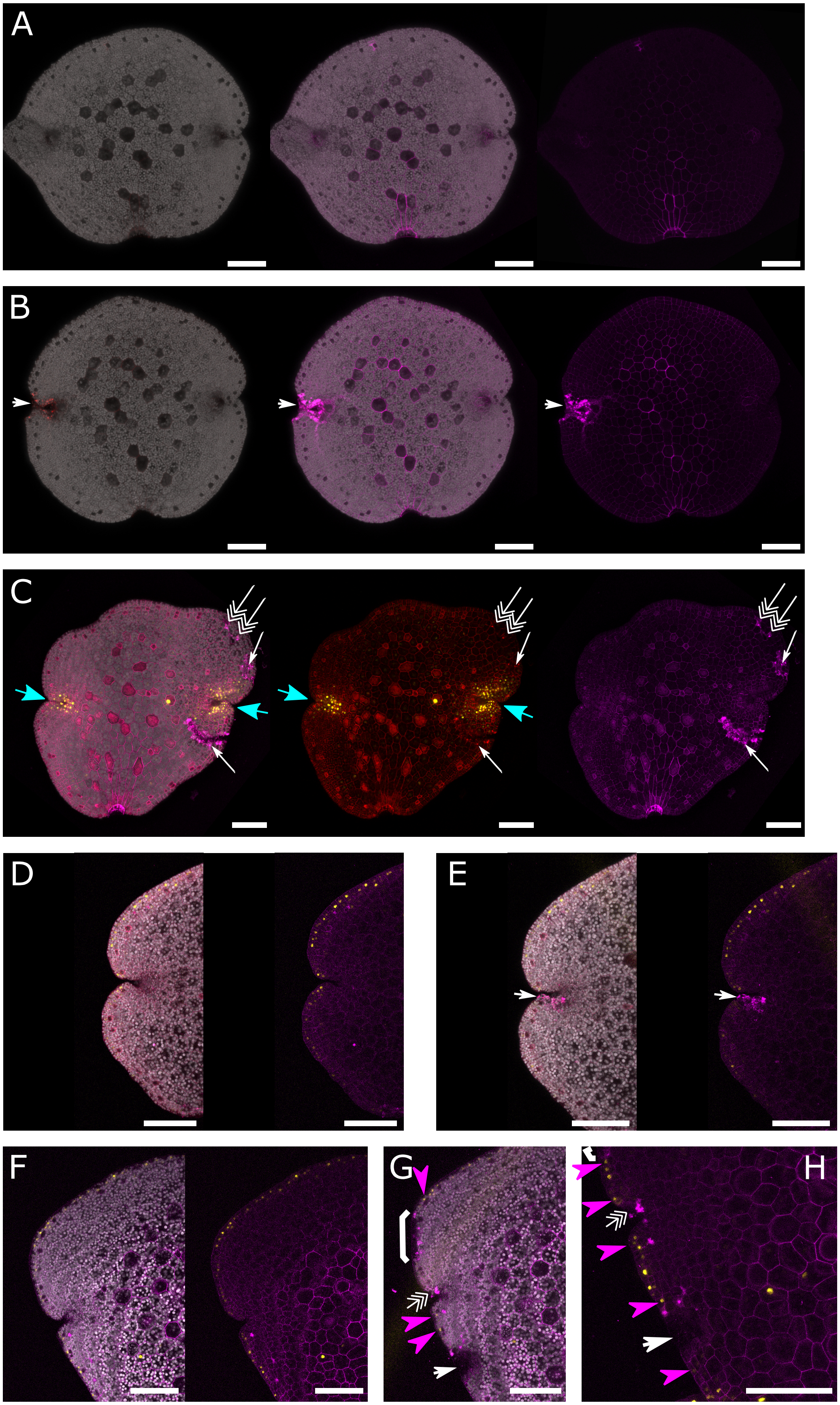
A
B
C
D
E
F
G
H

Fig. S11. Propidium iodide staining shows that laser ablation kills targeted cells without causing widespread cell death or stress in the gemma. Propidium iodide (PI) staining is shown in magenta. PI stains cell walls but does not penetrate the cell membranes of living cells, however upon cell death it goes into the remains of the cell, staining the nucleus with fluorescence emission in the mScarlet channel wavelengths. (A) shows an intact WT gemma, with no nuclear PI staining. (B) shows a WT gemma whose apical notch was subjected to laser ablation. Targeted cells have nuclear and intracellular PI staining (white arrows), demonstrating that this treatment effectively killed cells in the Marchantia gemma. The damage (also indicated by loss of chlorophyll autofluorescence and mechanical disruption) was restricted to the area targeted by the laser, with neighbouring cells and the wider gemma left intact. (C) shows a gemma from an enhancer trap line (ET239-P14) following various laser ablation targeting treatments. Regions targeted for tissue excision (white arrows) featured multiple other obvious signs of cell damage (loss of chlorophyll autofluorescence, membrane destruction and mechanical disruption) and there were no signs of widespread cell death or stress across the rest of the gemma. Ablation targeting single cells (triple white arrows) only killed those cells. Laser ablation treatment did not have off-target effects on apical notch/meristem mVenus marker signal (blue arrows). (D) shows a gemma region before ablation; (E) shows the same region afterwards. The lower side of the apical notch had been ablated (white arrows in (E)) with no damage caused to the other side of the apical notch. (F) shows a gemma region before ablation, (G) shows the same region after ablation and (H) is a close-up of the same post-ablation gemma. The ablation patterns used were a row of cells at the gemma edge (white brackets), targeting single cells (triple white arrows) and tissue excision (white arrows). Only the targeted areas showed cell death, with agreement between PI staining, loss of chlorophyll autofluorescence and mechanical disruption. Neighbouring cells were unaffected, and enhancer trap marker signal continued unaffected (magenta arrows). (D)-(H) shows gemmae from the enhancer trap line ET239-P64. (A) and (B) show, from left to right, the chlorophyll autofluorescence and mScarlet emssion channels overlaid; chlorophyll autofluorescence and PI emission channels overlaid; PI emission channel only. (C) shows, from left to right, chlorophyll autofluorescence, mScarlet, PI emission and mVenus channels overlaid; mScarlet and mVenus channels overlaid; PI emission channel only. (D)-(G) show, from left to right, chlorophyll autofluorescence, PI emission and mVenus channels overlaid; PI emssion and mVenus channels overlaid. (H) shows PI emssion and mVenus channels overlaid. Scale bars= 100µm.

Table S1. Excitation and collection wavelengths and settings used in confocal microscope imaging

Fluorophore	Excitation Wavelengths (nm)	Emission Detector Wavelength Tuning (nm)	Laser Intensity	Gain
mVenus	515 (White Light Laser)	522-541	60%	60
mScarlet	569 (White Light Laser)	589-625	40%	50
Chlorophyll	405 (Diode)	670-701	6%	20
Chlorophyll (SI Appendix Fig. S5)	488 (Diode)	650-700	0.06%	600V
Propidium Iodide	488, 535 (White Light Laser)	592 - 650	15%, 10%	40
Propidium Iodide (SI Appendix Fig. S5)	488nm (Diode)	585-617	3-4%	650V

Dataset S1 (separate file). Annotated sequence description of the plasmids used to generate the transgenic lines used in the experiments: L2_239-CsA (16,019 bp), L2_268-CsA (19,174 bp) and L2_283-CsA (26,315bp). The sequences and annotations are provided in Genbank format.

SI References

1. A. O. Marron, *et al.*, An Enhancer Trap System to study tissue development in *Marchantia polymorpha*. *Plant J.* **116**, 604–628 (2023).
